# TLR7/9 and type I interferon signaling reprogram neutrophil NET responses and impair antibacterial defense in systemic lupus erythematosus

**DOI:** 10.1016/j.cpblue.2026.100080

**Published:** 2026-07-23

**Authors:** Eden G. TenBarge, Ashley D. Wise, Morgan L. Hetzel, Helene A. Hoover, Helia Esfandiari, Bailey E. Holder, Eva Belevska, Ellie C. Mennen, Sarah R. McDaniel, Nicole M. Vaccaro, Courtney C. Lucca, Jonathan M. Williams, Jennifer Ferris, Timothy E. Sparer, Leslie J. Crofford, Jeffry D. Bieber, Andrew J. Monteith

**Affiliations:** 1 Department of Microbiology, University of Tennessee, Knoxville, TN, USA; 2 Division of Rheumatology, University of Tennessee Medical Center, Knoxville, TN, USA; 3 Division of Rheumatology and Immunology, Department of Medicine, Vanderbilt University Medical Center, Nashville, TN, USA; 4 Department of Pathology, Microbiology, and Immunology, Vanderbilt University Medical Center, Nashville, TN, USA; 5 Department of Biochemistry & Cellular and Molecular Biology, University of Tennessee, Knoxville, TN, USA; 6 Lead contact

## Abstract

Patients with systemic lupus erythematosus (SLE) are susceptible to bacterial infections, but the mechanisms remain unclear. We found that *Staphylococcus aureus* triggers mitochondria-dependent suicidal neutrophil extracellular trap (NET)osis through lactate sensing in healthy neutrophils, whereas this response is defective in SLE. Sustained Toll-like receptor (TLR) 7/9 signaling reduced mitochondrial lactate dehydrogenase B (LDHB), thereby impairing lactate sensing and suicidal NETosis. Instead, SLE neutrophils were biased toward vital NET release, a less bactericidal, type I interferon (IFN)-driven process initiated by staphylococcal pore-forming toxins and amplified by elevated systemic IFNα. In lupus-prone mice, combined hydroxychloroquine (HCQ) and interferon-alpha/beta receptor (IFNAR) blockade restored LDHB expression, rebalanced suicidal and vital NET responses, and improved bacterial clearance. Neutrophils from SLE patients showed similar defects that were reversed by HCQ and the IFNAR-blocking antibody anifrolumab. These findings define an immunometabolic checkpoint linking chronic autoimmune signaling to defective antibacterial defense in SLE and identify therapeutic routes to restore innate immunity.

## In brief

TenBarge et al. show that lupus-associated TLR7/9 and type I interferon signaling reprogram neutrophil NET responses during *Staphylococcus aureus* infection. This shifts neutrophils away from LDHB-dependent, bactericidal suicidal NETosis and toward less protective vital NET release, impairing antibacterial defense in lupus.

## Graphical Abstract

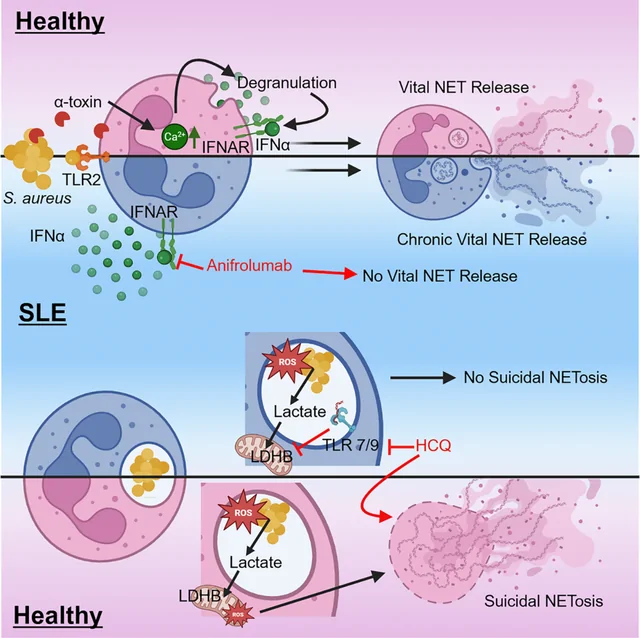

## INTRODUCTION

Neutrophils are critical for controlling microbial pathogens such as *Staphylococcus aureus*. However, their inflammatory responses must be tightly regulated to balance effective pathogen clearance with host tissue protection. A hallmark of this balance is the formation of neutrophil extracellular traps (NETs), which trap and kill *S. aureus*,^1–4^ prevent bacterial dissemination,^1,5^ and enhance macrophage activation.^6–8^ However, NETs can also drive pathology by exposing nuclear antigens and pro-inflammatory mediators, contributing to autoimmune responses.

In systemic lupus erythematosus (SLE), excessive NET deposition is widely reported and thought to perpetuate disease by promoting type I interferon (IFN) production,^9–11^ autoantibody generation,^12,13^ endothelial damage,^14–16^ thrombosis,^17,18^ and cardiovascular disease.^19,20^ Impaired NET clearance due to DNase deficiencies^21–23^ or defective lysosomal degradation by macrophages^24–26^ exacerbates these effects, prolonging local inflammation. Human studies similarly associate NET abundance with disease activity in SLE,^27–29^ suggesting that dysregulated NET formation and clearance are central to SLE pathology.

NETs are composed of decondensed chromatin decorated with antimicrobial proteins. Functionally, two mechanistically distinct forms exist: suicidal NETosis, in which NETs are released through lytic cell death,^2^ and vital NET release, where chromatin is extruded without compromising membrane integrity.^3^ Suicidal NETosis has historically been attributed to NADPH oxidase-derived reactive oxygen species (ROS),^30,31^ but recent studies highlighted a role for mitochondrial ROS as an essential trigger, particularly during responses to bacteria^7,32–34^ and in the autoimmune setting of SLE.^10,35^ In contrast, vital NET formation is ROS independent and can be triggered by microbial toxins^3^ or IFNα,^36^ although its regulation remains poorly understood. These two NET programs also differ kinetically, with vital NET release occurring rapidly and suicidal NETosis emerging over a longer time frame.^7,37^ Despite the differences in regulation, both suicidal and vital NET release require the citrullination of histones by protein arginine deiminase 4 (PAD4) to promote DNA decondensation.^38^

Despite their heightened tendency to release NETs, neutrophils from SLE patients fail to undergo mitochondrial ROS-dependent suicidal NETosis in response to *S. aureus*.^34,37^ Neutrophils are metabolically unique in that they rely primarily on glycolysis for energy,^39–43^ yet they retain functional mitochondria.^44,45^ We previously demonstrated that mitochondrial sensing of phagosomal lactate, a byproduct of *S. aureus* fermentation, acts as a critical danger signal that triggers mitochondrial ROS production and initiates suicidal NETosis.^34^ In SLE, however, neutrophils exhibit altered mitochondrial homeostasis that impairs this pathway, becoming biased toward sustained vital NET release.^34,37^ This paradox suggests that excessive NET formation in SLE may arise from dysregulated mechanisms that favor non-lytic, and potentially less effective, NET programs. This dysregulation may underlie the increased risk of severe bacterial infections in individuals with SLE.^46–49^ However, the signaling pathways linking chronic inflammation in SLE to altered neutrophil NET programs remain incompletely understood.

Here, we define how antibacterial NET responses are organized in healthy neutrophils and how this program is rewired in lupus. We show that TLR7/9 signaling suppresses mitochondrial lactate dehydrogenase B (LDHB), impairing lactate sensing and suicidal NETosis in response to *S. aureus*, while type I interferon signaling sustains a less bactericidal vital NET program. In lupus-prone mice, this altered NET balance is associated with impaired antibacterial defense and can be partially corrected by hydroxychloroquine (HCQ) and interferon-alpha/beta receptor (IFNAR) blockade. Importantly, neutrophils from SLE patients exhibit similar defects in LDHB expression and NET regulation, which are partially restored by HCQ and the IFNAR1-blocking antibody anifrolumab. Together, these findings link chronic autoimmune signaling to defective neutrophil antibacterial responses in SLE.

## RESULTS

### TLR signals differentially shape LDHB expression and suicidal NETosis in wild-type and lupus-prone neutrophils

We previously demonstrated that reverse conversion of staphylococcal lactate into pyruvate in the mitochondria is essential for triggering mitochondrial ROS and initiating suicidal NETosis.^34^ Mammalian cells express two distinct isoforms of LDH: LDHA, which catalyzes the conversion of pyruvate to lactate during glycolysis, and LDHB, which preferentially converts lactate back into pyruvate and associates with mitochondria.^50–52^ In line with this, we previously found that mitochondria from SLE neutrophils exhibit reduced LDH activity, specifically a diminished capacity to convert lactate to pyruvate, compared to healthy controls.^34^ Therefore, loss of LDHB in SLE neutrophils may impair sensing of phagosomal lactate from *S. aureus*, which would compromise suicidal NETosis.

**Figure 1.**
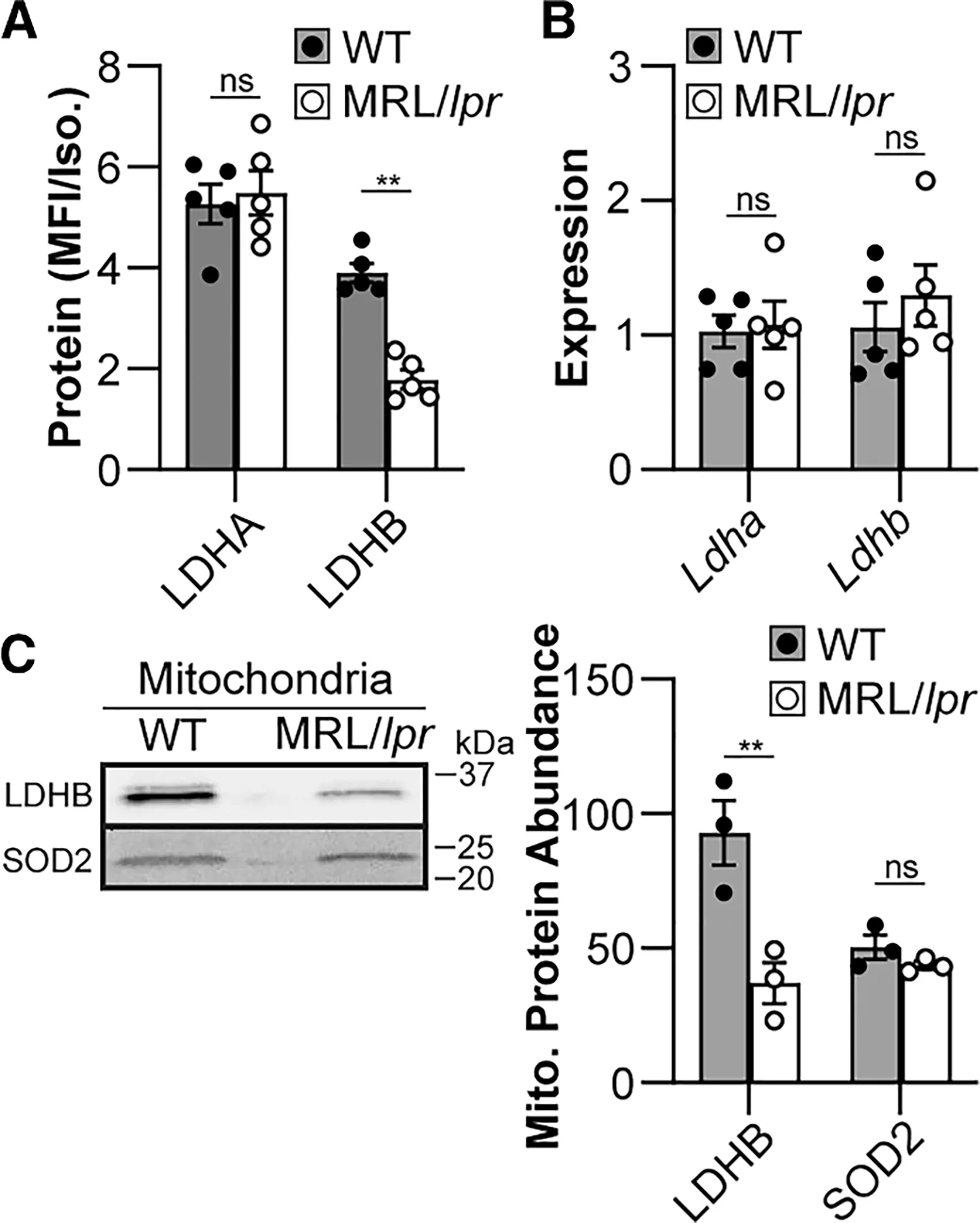
LDHB expression is suppressed in neutrophils from lupus-prone mice (A and B) Neutrophils were isolated from WT or MRL/*lpr*, and (A) total LDHA or LDHB protein were quantified by flow cytometry relative to an isotype control antibody or (B) *Ldha* or *Ldhb* transcript quantified relative to *Actb* by quantitative PCR. (C) Mitochondria were isolated from WT or MRL/*lpr* neutrophils and the abundance of LDHB or the mitochondrial superoxide dismutase 2 (SOD2) were quantified by immunoblot. Bands were quantified by integrated density. Each point represents neutrophils isolated from a single mouse. (A and B) Two-way ANOVA with Tukey multiple-comparisons test or (C) paired *t* test; error bars reflect standard error (**p ≤ 0.01; ns, not significant).

To investigate this, we used MRL/MpJ-*Tnfrs6*^lpr^/J (MRL/*lpr*) mice, a well-established murine model of SLE that recapitulates key autoimmune features of the human disease.^53^ Neutrophils were isolated from MRL/*lpr* and wild type (WT) C57BL/6 mice, and expression of LDHA and LDHB were assessed by flow cytometry (Figure S1A). MRL/*lpr* neutrophils expressed significantly lower levels of LDHB compared to WT, whereas LDHA levels remained unchanged (Figures 1A and S1B). *Ldhb* mRNA levels were comparable between WT and MRL/*lpr* neutrophils (Figure 1B), suggesting that LDHB is regulated post-transcriptionally. Consistent with this, mitochondrial fractions from MRL/*lpr* neutrophils also showed reduced LDHB protein abundance by immunoblot (Figure 1C), indicating loss of mitochondrial LDHB.

**Figure 2.**
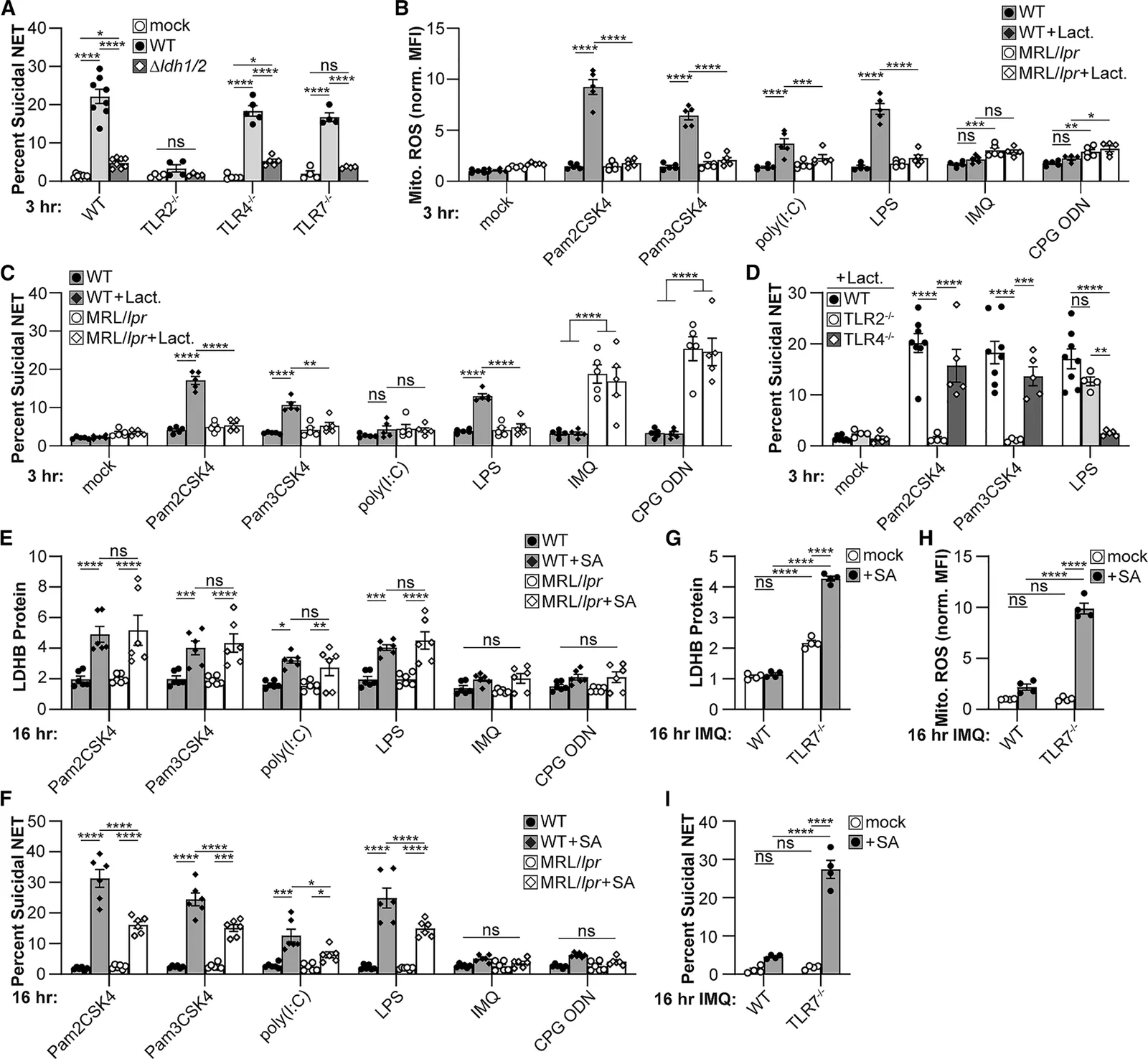
TLR signals differentially shape LDHB expression and suicidal NETosis in WT and lupus-prone neutrophils (A) WT, TLR2^−/−^, TLR4^−/−^, or TLR7^−/−^ neutrophils were cultured with WT or Δ*ldh1/2 S. aureus* for 3 h and suicidal NETosis quantified by flow cytometry (see Figures S1A–S1D for gating strategy and marker definitions). (B–D) WT, TLR2^−/−^, TLR4^−/−^, or MRL/*lpr* neutrophils were treated with differing TLR agonists in the presence or absence of lactate (1 mM) and (B) mitochondrial ROS or (C and D) suicidal NETosis were quantified by flow cytometry. (E–I) Neutrophils were cultured for 16 h with IFNγ (50 ng/mL) and the indicated TLR agonist (50 ng/mL) under prolonged *ex vivo* priming conditions prior to *S. aureus* challenge. Neutrophils were challenged with *S. aureus* (SA) for 3 h and (E and G) total LDHB protein relative to an isotype control antibody, (H) mitochondrial ROS, or (F and I) suicidal NETosis were quantified by flow cytometry. Each point represents neutrophils isolated from a single mouse. Two-way ANOVA with Tukey multiple-comparisons test; error bars reflect standard error (*p ≤ 0.05, **p ≤ 0.01, ***p ≤ 0.001, ****p ≤ 0.0001; ns, not significant).

While we previously identified staphylococcal lactate as a trigger for mitochondrial ROS-dependent suicidal NETosis,^34^ we had not directly tested whether TLR engagement was required for this process. To address this, we stimulated WT neutrophils or neutrophils lacking TLR2, TLR4, or TLR7 with WT *S. aureus* or a strain that cannot make L-lactate (Δ*ldh1/2*). Neutrophils lacking TLR2 failed to undergo suicidal NETosis in response to *S. aureus*, and the Δ*ldh1/2* strain elicited reduced suicidal NETosis (Figure 2A), consistent with our prior findings.^34^ These results indicate that both TLR2 engagement and bacterial lactate production are required to trigger mitochondrial ROS-dependent suicidal NETosis.

We previously demonstrated that WT neutrophils undergo mitochondrial ROS-dependent suicidal NETosis in response to *S. aureus*, whereas MRL/*lpr* neutrophils instead undergo suicidal NETosis in response to apoptotic debris, suggesting distinct and potentially antagonistic NET programs.^34^ To explore upstream drivers of this divergence, we stimulated WT and MRL/*lpr* neutrophils with agonists for TLR2/1, TLR2/6, TLR3, TLR4, TLR7, and TLR9 in the presence or absence of exogenous lactate. WT neutrophils generated robust mitochondrial ROS (Figures 2B and S1C) in response to TLR2 and TLR4 agonists when lactate was present. To quantify NET formation, we used a flow cytometry-based technique that has been previously described in detail^7^ (Figure S1D). Using this assay framework, *S. aureus* induces both vital and suicidal NET responses with distinct kinetics, such that vital NET release predominates at early time points whereas suicidal NETosis becomes more prominent later.^7,37^ Stimulation with TLR2 and TLR4 agonists in the presence of exogenous lactate triggered neutrophils to undergo suicidal NETosis (Figures 2C and S1E), consistent with their role in bacterial sensing. Neutrophils deficient in TLR2 or TLR4 did not undergo suicidal NETosis upon stimulation with their cognate ligands in the presence of lactate, confirming pathway specificity (Figure 2D). In contrast, MRL/*lpr* neutrophils responded to TLR7 and TLR9 agonists with lactate-independent suicidal NETosis (Figures 2B and 2C; Figures S1D and S1E), indicating a shift in NETosis programming.

Previous work in LCMV-infected dendritic cells demonstrated that prolonged TLR7 activation can reduce LDHB expression.^54^ Given that nuclear antigens accumulate in SLE and activate endosomal TLRs^55,56^ including TLR7, we next asked whether defined inflammatory conditioning through different TLR pathways altered LDHB expression and subsequent NETosis in response to *S. aureus*. WT and MRL/*lpr* neutrophils were cultured for 16 h with TLR agonists in the presence of IFNγ (50 ng per mL each) using a prolonged *ex vivo* priming protocol^57^ that largely maintains neutrophil viability (Figure S1F). Because neutrophils did not survive well overnight with IFNγ alone, the prolonged culture assay required both IFNγ and a TLR agonist to maintain sufficient viability for subsequent *S. aureus* challenge. We also note that prolonged culture altered the baseline LDHB relationship observed in freshly isolated WT and MRL/*lpr* neutrophils (Figure 1A). Therefore, we did not interpret this assay relative to an unstimulated 16-h baseline. Instead, we used it to compare how different inflammatory priming conditions altered LDHB expression, mitochondrial ROS, and suicidal NETosis after subsequent *S. aureus* challenge.

Under these conditions, WT neutrophils cultured with TLR2 or TLR4 agonists exhibited higher LDHB expression (Figures 2E and S1G) and greater suicidal NETosis (Figures 2F and S1H) after subsequent *S. aureus* challenge than WT neutrophils cultured with TLR7 or TLR9 agonists. Similarly, in MRL/*lpr* neutrophils, prolonged culture with TLR2 or TLR4 agonists restored LDHB expression to levels comparable to those of WT neutrophils and partially rescued *S. aureus*-induced suicidal NETosis relative to the WT response, whereas TLR7 or TLR9 conditioning did not produce the same rescue (Figures 2E and 2F). To test whether the IMQ-associated phenotype required TLR7, we performed parallel experiments using TLR7-deficient neutrophils. Although IMQ may have TLR7-independent effects during prolonged culture, TLR7-deficient neutrophils maintained LDHB expression (Figure 2G), mitochondrial ROS (Figure 2H), and suicidal NETosis (Figure 2I) following IMQ treatment and *S. aureus* challenge. These findings indicate that the effect of IMQ on the LDHB-associated suicidal NET response requires TLR7.

Together, these findings indicate that distinct TLR-dependent priming conditions differentially shape LDHB-associated mitochondrial responses and suicidal NETosis following subsequent *S. aureus* challenge. TLR2/4-skewed conditioning favored an LDHB-high, suicidal NET-competent response, whereas TLR7/9-skewed conditioning produced lower LDHB expression and weaker suicidal NETosis relative to the TLR2/4-skewed condition.

### HCQ restores *S. aureus*-induced suicidal NETosis but MRL/*lpr* neutrophils have impaired antibacterial activity

**Figure 3.**
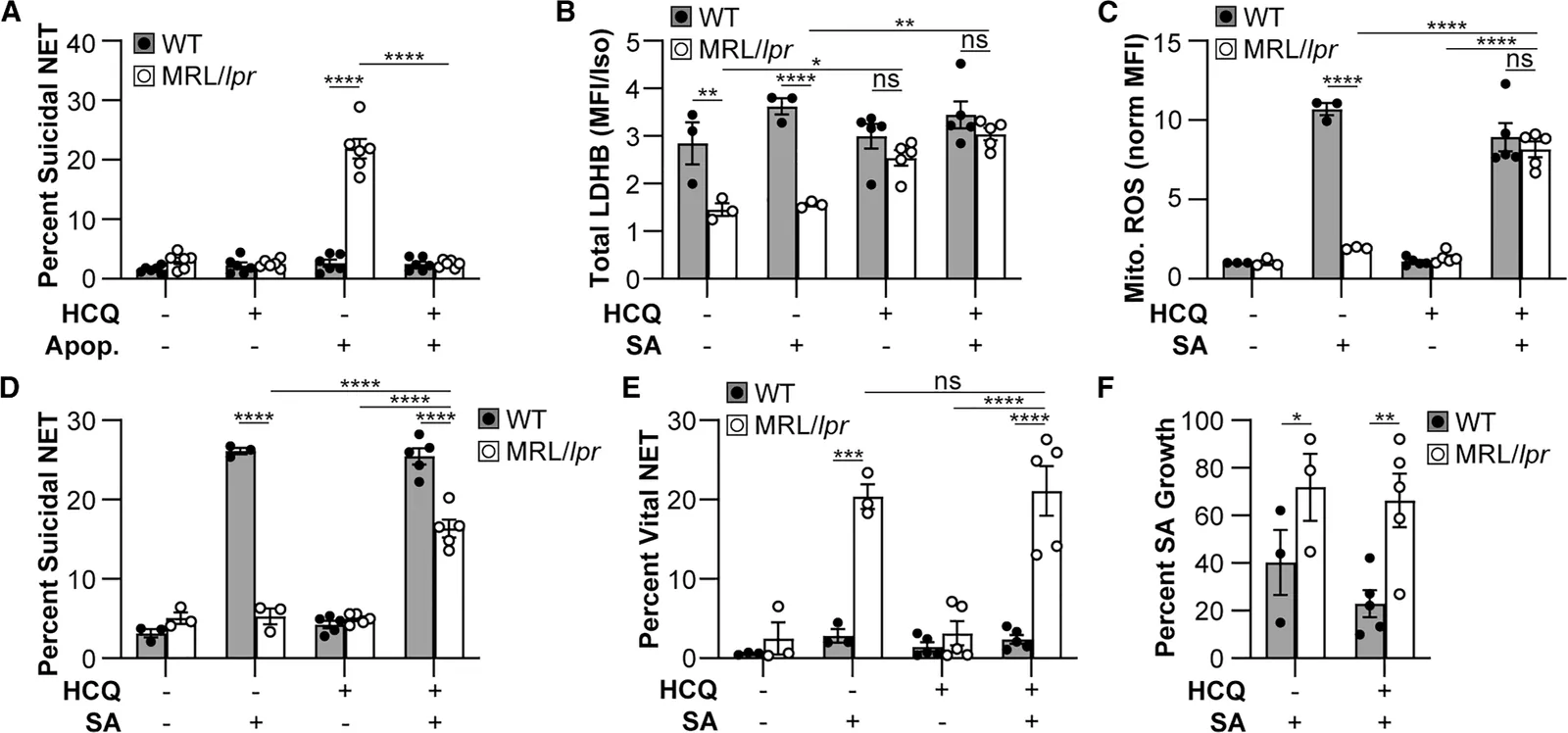
HCQ restores *S. aureus*-induced mitochondrial ROS and suicidal NETosis but does not fully rescue bactericidal activity in MRL/*lpr* neutrophils (A) WT or MRL/*lpr* neutrophils were treated with HCQ (30 μM) and subsequently stimulated with apoptotic debris. After 3 h, suicidal NETosis was quantified by flow cytometry. (B–F) WT or MRL/*lpr* mice were mock treated or treated with HCQ (40 mg/kg) daily for 2 days. Neutrophils were isolated from the bone marrow, cultured with *S. aureus* (SA) for 3 h, and (B) total LDHB protein relative to an isotype control, (C) mitochondrial ROS, and neutrophils undergoing (D) suicidal NETosis or (E) vital NET release were quantified by flow cytometry. (F) Neutrophils were cultured with *S. aureus* for 4 h, and bacterial killing relative to *S. aureus* grown in the absence of neutrophils was quantified by dilution spot plating. Each point represents neutrophils isolated from a single mouse. Two-way ANOVA with Tukey multiple-comparisons test; error bars reflect standard error (*p ≤ 0.05, **p ≤ 0.01, ***p ≤ 0.001, ****p ≤ 0.0001; ns, not significant).

HCQ, commonly prescribed as Plaquenil, is an acidotropic molecule that binds double- and single-stranded nucleotides and sterically hinders their binding to TLR7/9.^58^ Clinically, HCQ is approved for the treatment of SLE and reduces disease flares by dampening type I interferon responses. We previously demonstrated that apoptotic debris triggers aberrant suicidal NETosis in SLE neutrophils.^34^Treating MRL/*lpr* neutrophils with HCQ prevented this spontaneous NET release in response to apoptotic stimuli (Figure 3A).

To determine whether HCQ also restores the bacterially induced suicidal NETosis in response to *S. aureus*, WT and MRL/*lpr* mice were treated with HCQ every 24 h for 2 days. This short treatment regimen was not intended to model the full therapeutic time course of HCQ in lupus, but rather to test whether brief HCQ exposure could rapidly alter neutrophil-intrinsic signaling and protein expression. Neutrophils were then isolated and stimulated *ex vivo* with *S. aureus*. HCQ treatment in MRL/*lpr* mice restored LDHB expression to levels comparable to WT (Figures 3B and S2A), increased mitochondrial ROS generation in response to *S. aureus* (Figures 3C and S2B), and re-established the capacity to undergo suicidal NETosis (Figure 3D; Figures S2C and S2D).

Despite this restoration of mitochondrial programming, a large subset of MRL/*lpr* neutrophils continued to undergo constitutive vital NET release (Figures 3E and S2E), a response previously shown to be less effective at bacterial killing.^37^ Consistent with this, MRL/*lpr* neutrophils from HCQ-treated mice remained deficient in controlling *S. aureus* infection *ex vivo*, exhibiting impaired bacterial clearance relative to WT controls (Figure 3F). These findings demonstrate that HCQ treatment can partially rescue suicidal NETosis by restoring LDHB expression and mitochondrial ROS production. However, MRL/*lpr* neutrophils still exhibit sustained vital NET release and remain functionally compromised in their antibacterial response, suggesting that restoration of the LDHB-dependent suicidal NET pathway is not sufficient to fully reverse the predisposition toward vital NET release in lupus-prone neutrophils. These data suggest that restoring mitochondrial signaling is necessary but not sufficient for effective bacterial clearance in SLE, potentially due to a persistent bias toward vital NET formation.

### Vital NET release is ineffective at killing *S. aureus* and interferes with the suicidal NETosis

Although HCQ treatment restores mitochondrial ROS and partially rescues suicidal NETosis in MRL/*lpr* neutrophils, these cells continue to exhibit constitutive vital NET release, suggesting that persistence of this program may limit full restoration of the antibacterial suicidal NET response. We therefore hypothesized that vital NET release is intrinsically less bactericidal than suicidal NETosis and may actively suppress the latter, either by depleting cellular resources or enforcing a distinct regulatory program.

**Figure 4.**
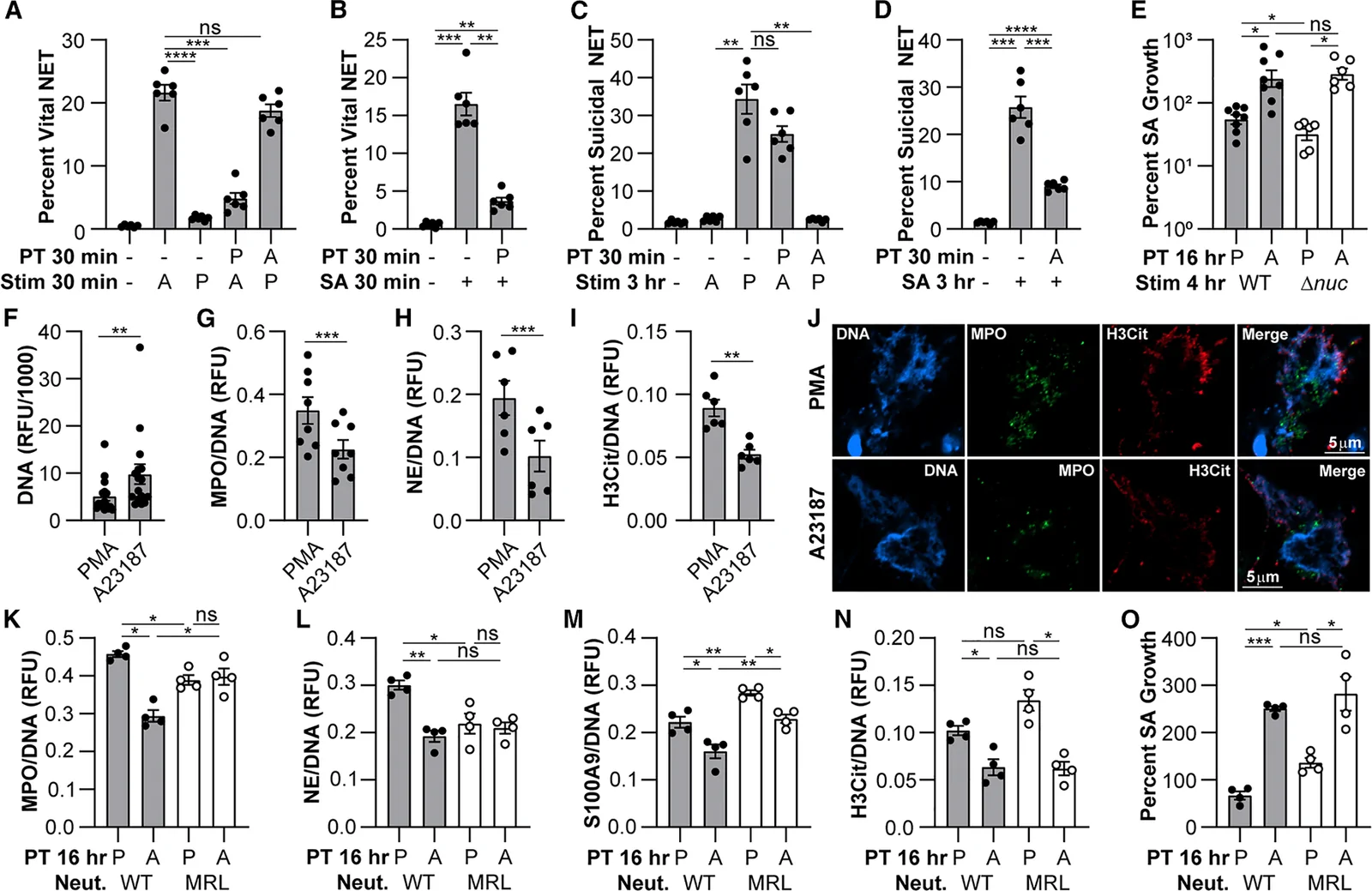
Vital and suicidal NET programs are functionally antagonistic and differ in antibacterial potency and composition (A–D) WT neutrophils were pretreated (PT) with PMA (P; 5 μM) or A23187 (A; 50 nM) and subsequently stimulated with PMA, A23187, or *S. aureus* (SA). (A and B) Vital NET release after 30 min or (C and D) suicidal NETosis after 3 h were quantified by flow cytometry. (E–O) WT and MRL/*lpr* neutrophils were treated with PMA or A23187 for 16 h and the resulting NETs subsequently (E and O) cultured with WT or Δ*nuc S. aureus* for 4 h. Bacterial killing was quantified relative to *S. aureus* cultured in the absence of NETs by dilution spot plating. (F–N) Alternatively, NETs were stained to quantify (F) DNA, (G and K) MPO, (H and L) NE, (M) S100A9, or (I and N) H3Cit using a fluorescent plate reader or (J) confocal microscopy (bar, 5 μm). Each point represents (A–D) neutrophils isolated from a single mouse or (E–I and K–O) NETs from neutrophils isolated from a single mouse. (A–E and K–O) One-way ANOVA with Tukey multiple-comparisons test or (F–I) paired *t* test; error bars reflect standard error (*p ≤ 0.05, **p ≤ 0.01, ***p ≤ 0.001, ****p ≤ 0.0001; ns, not significant).

To experimentally model how one NET program may influence another, we established an *in vitro* system to sequentially activate vital and suicidal NET pathways. Neutrophils were pretreated with A23187, a calcium ionophore that triggers rapid, ROS-independent vital NET release,^59^ followed by stimulation with phorbol myristate acetate (PMA), which induces ROS-dependent suicidal NETosis,^2,59^ or *S. aureus*, a biologically relevant stimulus that induces both vital^3^ and suicidal NET responses,^2^ but with distinct kinetics: vital NET release occurs rapidly, whereas suicidal NETosis becomes more prominent at later time points.^7^ A23187 pretreatment led to robust vital NET release (Figures 4A and 4B) but significantly reduced the fraction of neutrophils undergoing subsequent suicidal NETosis in response to PMA or *S. aureus* (Figures 4C and 4D). Conversely, pretreatment with PMA induced suicidal NETosis (Figures 4C and 4D) and reduced subsequent vital NET release in response to A23187 and *S. aureus* (Figures 4A and 4B). These findings indicate that prior engagement of one NET program can render neutrophils less responsive to induction of the alternate program, consistent with early commitment to distinct NET states.

To assess whether these NET programs differ in antibacterial potency, neutrophils were stimulated with A23187 or PMA and cultured for 16 h to allow NET formation. A23187-induced NETs exhibited significantly reduced capacity to kill *S. aureus* compared to PMA-induced suicidal NETs (Figure 4E). This difference in bactericidal activity was not due to preferential degradation of suicidal NETs by staphylococcal nucleases, as a nuclease-deficient staphylococcal strain (Δ*nuc*) remained equally resistant to A23187-induced NETs. Despite their impaired antibacterial function, A23187 stimulation led to more abundant NET deposition than PMA (Figure 4F). However, these vital NETs contained less antimicrobial proteins such as myeloperoxidase (MPO) and elastase (NE), as well as reduced citrullinated histones (H3Cit; Figures 4G–4J). Together, these results demonstrate that vital NETs are both less bactericidal and capable of antagonizing suicidal NETosis.

To further dissect how NET subtype differences contribute to antibacterial efficacy, we compared NET composition and function between WT and MRL/*lpr* neutrophils following stimulation with PMA or A23187. Both genotypes released similar amounts of NETs (Figure S3A), but their composition diverged. Suicidal NETs from MRL/*lpr* neutrophils contained lower levels of MPO and NE but elevated calprotectin (S100A9) compared to WT (Figures 4K–4M), while vital NETs exhibited modest increases in MPO and calprotectin with similar NE levels. Citrullinated histone content was comparable in vital and suicidal NETs between genotypes (Figure 4N). Suicidal NETs from MRL/*lpr* neutrophils were less bactericidal than WT (Figure 4O), consistent with reduced MPO and NE content (Figures 4K–4L). In contrast, vital NETs were comparably ineffective at killing *S. aureus* in both genotypes (Figure 4O), despite differences in MPO and calprotectin content (Figures 4K and 4M). These findings suggest that the reduced antibacterial capacity of MRL/*lpr* neutrophils reflects both impaired suicidal NET function and a disproportionate reliance on vital NET release, a NET program that is intrinsically less bactericidal.

### Staphylococcal pore-forming toxins cause calcium influx and vital NET release

**Figure 5.**
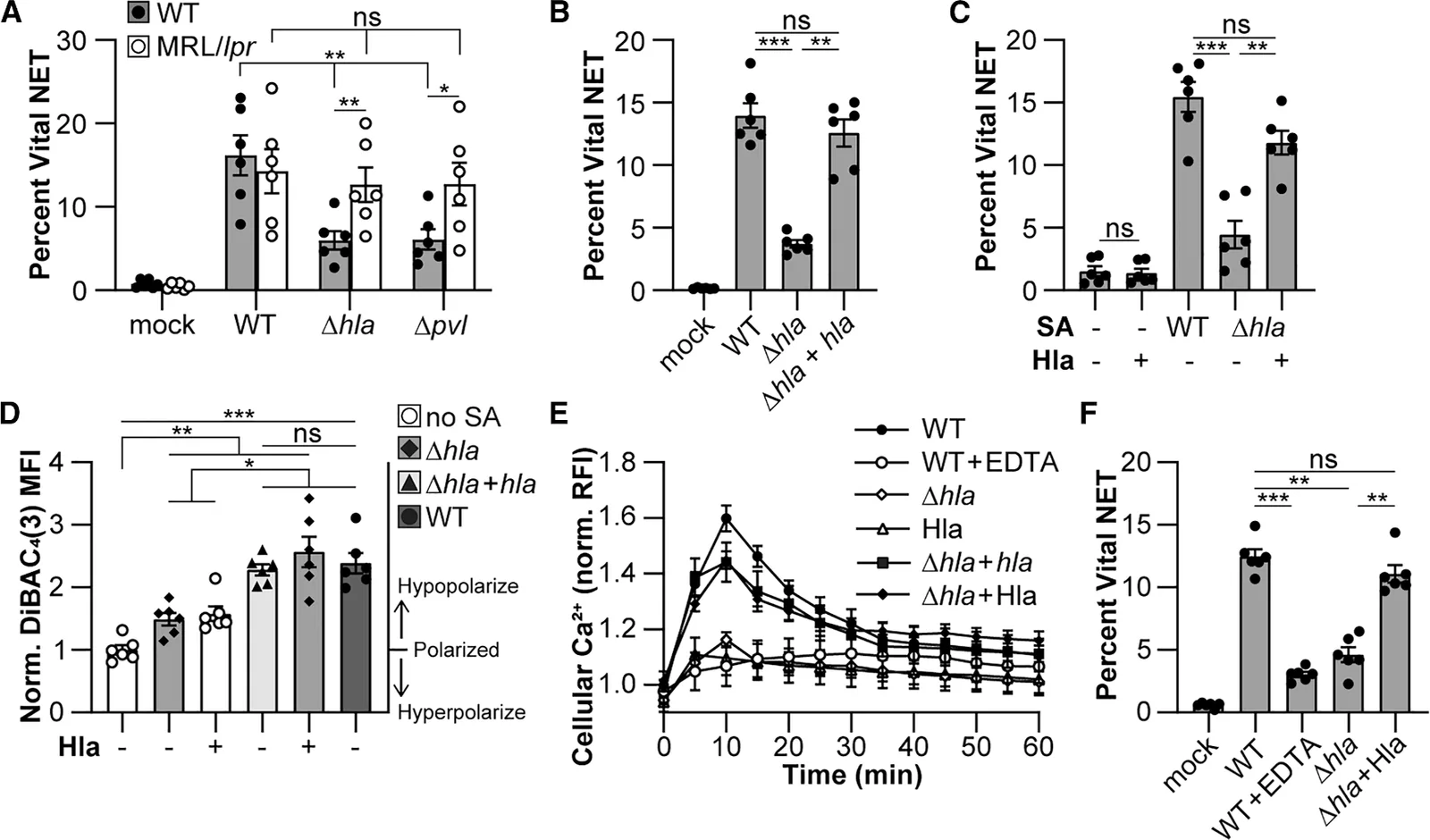
Staphylococcal Hla promotes vital NET release by inducing calcium influx in neutrophils (A–C) WT or MRL/*lpr* neutrophils were cultured with WT, Δ*hla*, Δ*pvl,* or *Δhla* + *hla S. aureus* in the presence or absence of Hla protein, and vital NET release was quantified after 30 min by flow cytometry. (D) WT neutrophils were loaded with DiBAC_4_(3) and stimulated with Δ*hla*, *Δhla* + *hla*, and/or Hla protein. The median fluorescence intensity (MFI) was quantified by flow cytometry and normalized to unstimulated neutrophils. (E) WT neutrophils were loaded with Fluo-4 and Hoechst 33342 and stimulated with WT, Δ*hla*, or *Δhla* + *hla S. aureus* in the presence or absence of EDTA (0.5 mM) or Hla protein (1 μM). Fluo-4 and Hoechst 33342 fluorescence was quantified temporally using a fluorescence plate reader and normalized to unstimulated neutrophils. (F) WT neutrophils were cultured with WT or Δ*hla S. aureus* in the presence of EDTA or Hla protein. Vital NET release was quantified by flow cytometry. Each point represents (A–D and F) neutrophils isolated from a single mouse or (E) the mean normalized (Fluo-4/Hoechst 33342) relative fluorescence intensity (RFI) from neutrophils isolated from 5 mice. (A) Two- or (B–D and F) one-way ANOVA with Tukey multiple-comparisons test; error bars reflect standard error (*p ≤ 0.05, **p ≤ 0.01, ***p ≤ 0.001; ns, not significant).

While the molecular regulation of suicidal NETosis is well characterized, the signaling pathways that drive vital NET release remain poorly defined. Prior work has shown that the pore-forming toxin Panton-Valentine leukocidin (PVL), secreted by *S. aureus*, can trigger vital NET release.^3^ To expand on this, we cultured neutrophils with *S. aureus* mutants lacking either PVL (Δ*pvl*) or α-hemolysin (Δ*hla*), another pore-forming toxin. Both mutants induced significantly less vital NET release compared to WT *S. aureus* (Figure 5A), confirming that these toxins contribute to vital NET release in WT neutrophils. In contrast, MRL/*lpr* neutrophils released vital NETs independent of these toxins, consistent with their dysregulated NET program.

Although PVL promotes vital NET release, we excluded it from further experiments due to its capacity to trigger mitochondrial ROS and suicidal NETosis,^33^ which could confound downstream analysis. The Δ*hla* strain did not affect NETosis (Figure S3B), and thus became the focus of subsequent studies. Expressing Hla in *trans* (Figure 5B) or adding exogenous Hla protein (Figures 5C and S3C) restored vital NET release by WT neutrophils exposed to the Δ*hla* strain. However, Hla protein alone was insufficient to trigger NET release, indicating that it acts in synergy with other bacterial factors. Together, these data demonstrate that *S. aureus* pore-forming toxins, including Hla, are key inducers of vital NET release during infection.

Neutrophils are relatively resistant to Hla-mediated lysis, suggesting that Hla may have immunomodulatory functions beyond cytotoxicity. Differing studies have produced contrasting results where Hla permits calcium influx^60^ or causes depolarization of the cell membrane and prevented calcium influx^61^ leading to enhanced neutrophil inflammatory responses^60,62–64^ but impaired chemotactic migration.^61,65^ To test whether Hla alters membrane potential, we loaded neutrophils with the voltage-sensitive dye DiBAC4(3) and stimulated them with differing *S. aureus* strains and/or Hla protein. Each treatment increased DiBAC4(3) fluorescence, indicating membrane depolarization, but either genetic complementation of the Δ*hla* strain or addition of exogenous Hla protein caused a further increase beyond either treatment alone and approximated the response observed with WT *S. aureus* (Figures 5D and S3D). This suggests that Hla induces depolarization, likely via ion flux, and that co-stimulation with *S. aureus* further amplifies depolarization.

Because prior work also implicated Hla in calcium signaling,^61^ we tested whether *S. aureus* triggers calcium influx. Neutrophils loaded with the calcium-sensitive dye Fluo-4 showed a rapid and robust increase in intracellular calcium in response to WT *S. aureus*, which was markedly reduced in cells treated with the Δ*hla* strain or calcium chelator EDTA (Figure 5E). Hla protein alone did not elicit calcium influx, but genetic complementation or exogenous Hla restored calcium flux in response to Δ*hla* to levels comparable to WT, indicating that Hla promotes calcium entry only in the context of bacterial stimulation. Furthermore, vital NET release was diminished when WT neutrophils were stimulated with the Δ*hla* mutant or treated with EDTA (Figure 5F), underscoring the requirement for both calcium influx and pore-forming toxins. These findings support a model in which Hla-dependent pore formation enables calcium entry into neutrophils, triggering vital NETosis during *S. aureus* infection.

### IFNα sustains vital NET release in MRL/*lpr* neutrophils responding to *S. aureus*

**Figure 6.**
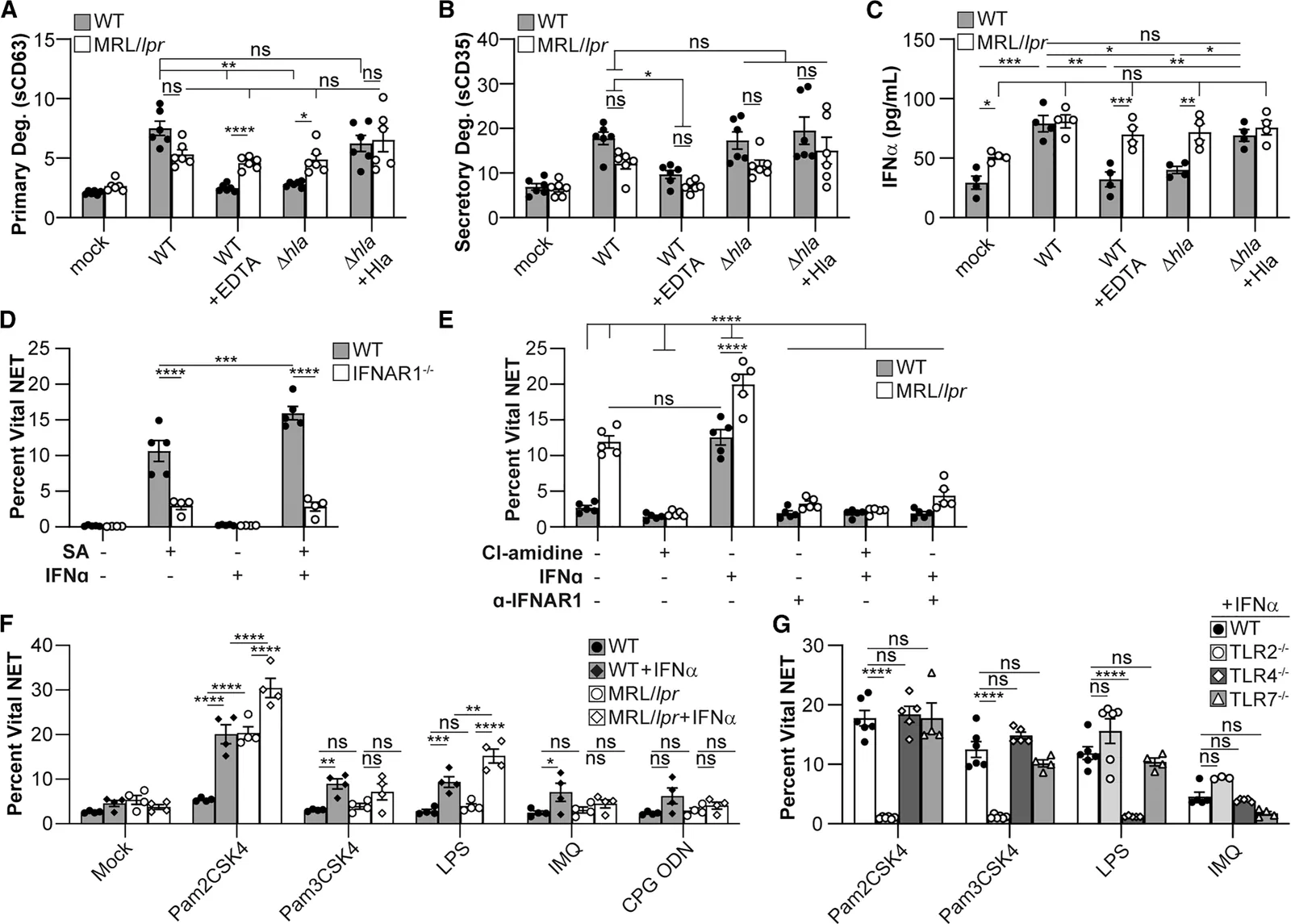
IFNα secretion coincides with calcium-dependent degranulation and is dysregulated in lupus-prone neutrophils (A–D) WT or MRL/*lpr* neutrophils were cultured with WT or Δ*hla S. aureus* in the presence or absence of EDTA or Hla protein. After 30 min, neutrophils were stained for surface expression of (A) CD63 (primary granules) or (B) CD35 (secretory granules) and quantified by flow cytometry or (C) supernatants isolated to quantify IFNα by ELISA. (D) WT or IFNAR1^−/−^ neutrophils were cultured with *S. aureus* and/or IFNα for 30 min and vital NET release quantified by flow cytometry. (E–G) WT or MRL/*lpr* neutrophils were (E) treated with Cl-amidine to broadly inhibit NET release, IFNα, and/or an α-IFNAR1 antibody and cultured with *S. aureus* or (F and G) treated with TLR agonists in the presence or absence of IFNα. After 3 h, vital NET release was quantified by flow cytometry. Each point represents neutrophils isolated from a single mouse. Two-way ANOVA with Tukey multiple-comparisons test; error bars reflect standard error (*p ≤ 0.05, **p ≤ 0.01, ***p ≤ 0.001, ****p ≤ 0.0001; ns, not significant).

IFNα has been shown to induce vital NET release in response to pathogens such as *Mycobacterium tuberculosis*.^36^ Given that vital NETs are released within minutes of *S. aureus* stimulation,^3^ we hypothesized that IFNα-driven signaling in this context relies on the rapid secretion of preformed protein rather than *de novo* transcription or translation. Specifically, we posited that calcium influx triggers degranulation and release of IFNα stores.^66–68^ To test this, neutrophils were stimulated with WT or Δ*hla S. aureus*, and surface expression of CD63 and CD35 was quantified by flow cytometry as markers of primary and secretory degranulation, respectively. Primary degranulation in response to *S. aureus* was dependent on the presence of Hla and calcium as the Δ*hla* strain did not trigger and EDTA prevented release of primary granules (Figures 6A and S3E). In contrast, MRL/*lpr* neutrophils released primary granules in response to *S. aureus* independent of Hla or calcium availability. As an orthogonal measure of primary granule release, we also quantified NE in culture supernatants, which showed the same dependencies in WT and MRL/*lpr* neutrophils (Figure S3F). Secretory degranulation in response to *S. aureus* was dependent solely on the presence of calcium as the Δ*hla* strain triggered release of secretory granules (Figure 6B). These findings indicate that *S. aureus*-induced degranulation is differentially regulated: primary granule release requires both Hla and calcium, while secretory granule release depends only on calcium.

To determine whether IFNα is released during degranulation, we quantified IFNα secretion by ELISA. WT neutrophils released IFNα in response to WT *S. aureus*, but not to the Δ*hla* strain (Figure 6C). This defect was rescued by adding Hla protein, confirming that Hla is required for IFNα release. However, calcium chelation with EDTA abolished IFNα secretion, indicating that release is calcium dependent. This pattern mirrored primary granule degranulation and suggests that IFNα is either stored in primary granules or released concurrently with them. In contrast, MRL/*lpr* neutrophils secreted IFNα regardless of Hla or calcium (Figure 6C), consistent with their constitutive, dysregulated NET phenotype. Together, these results support a model in which staphylococcal pore-forming toxins initiate IFNα release that drives vital NET release, a pathway that is uncoupled or rendered constitutively active in lupus-prone neutrophils.

To determine whether IFNα drives vital NET release, WT and IFNAR1^−/−^ neutrophils were stimulated with *S. aureus* in the presence or absence of recombinant IFNα. IFNAR1^−/−^ neutrophils were unable to undergo vital NET release in response to *S. aureus*, demonstrating that IFNAR signaling is essential for this pathway (Figure 6D). Recombinant IFNα further enhanced NET release in WT neutrophils but had no effect on IFNAR1^−/−^ cells, confirming that IFNα acts directly through IFNAR to promote vital NETosis. These findings place IFNα downstream of Hla-dependent degranulation and identify IFNAR signaling as a requisite amplifier of vital NET formation.

We next tested whether IFNα sustains chronic vital NET release. MRL/*lpr* neutrophils released more vital NETs than WT neutrophils after 3 h of *S. aureus* stimulation (Figures 6E and S3G), consistent with prior studies.^37^ Recombinant IFNα enhanced NET release in both WT and MRL/*lpr* neutrophils, while IFNAR1 blockade or PAD4 inhibition suppressed it. However, IFNα alone was insufficient as TLR co-stimulation was required. In TLR agonist assays, IFNα enhanced NETosis only when paired with a TLR ligand, except in MRL/*lpr* neutrophils responding to TLR2/1, which released NETs even without IFNα (Figure 6F). To confirm the specificity of this response, neutrophils deficient in TLR2 and TLR4 did not undergo vital NET release upon stimulation with their cognate ligands and IFNα (Figure 6G). These results suggest that TLR2/1 signaling bypasses IFNα dependence in lupus-prone neutrophils and that IFNAR-PAD4 signaling integrates with bacterial sensing to sustain vital NETosis. Overall, our findings support a model in which Hla triggers calcium-dependent IFNα release, which then sustains NET release via IFNAR and PAD4, a program that becomes dysregulated in lupus.

**Figure 7.**
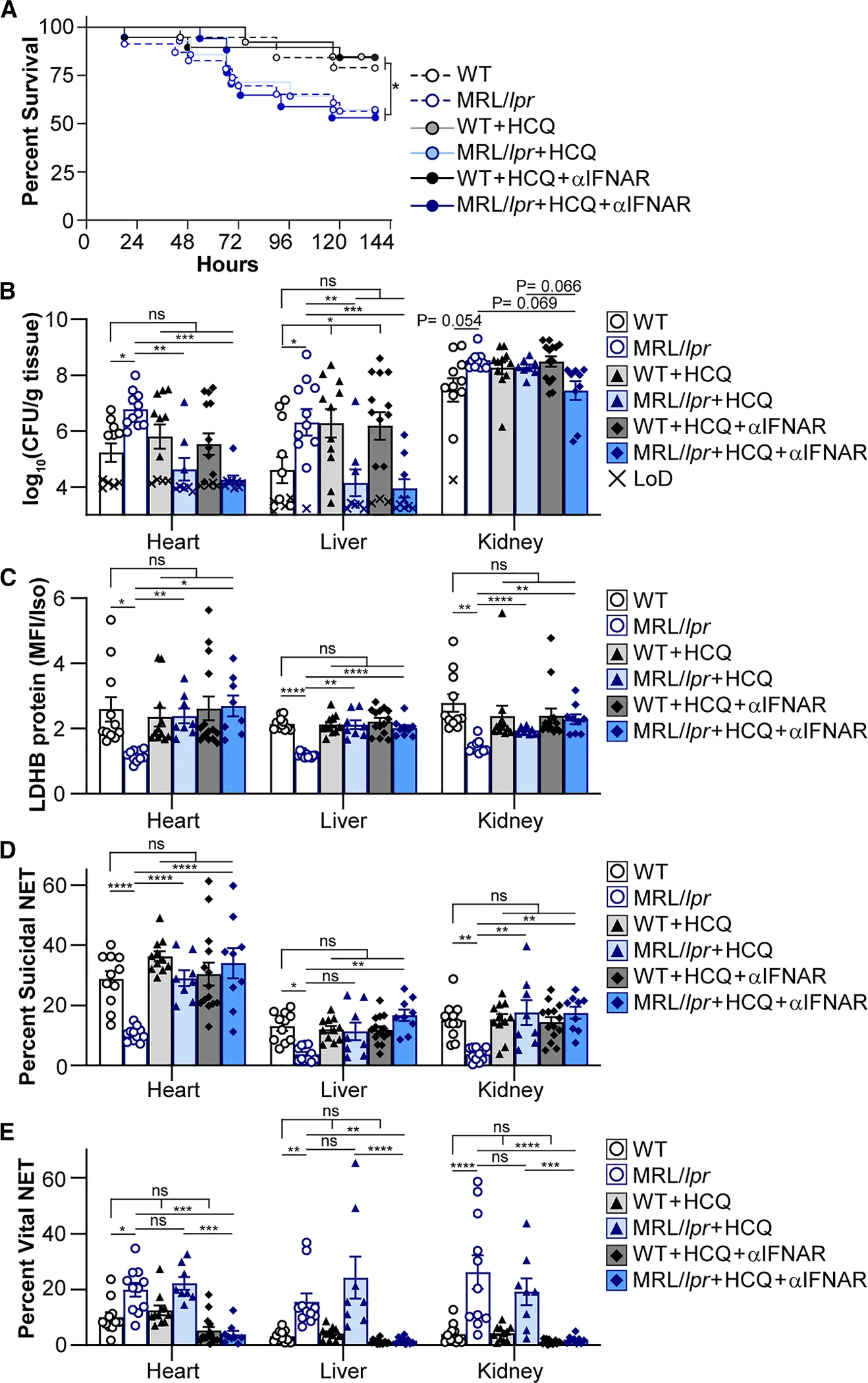
Combined HCQ and IFNAR1 blockade restores NET subtype balance and antibacterial defense in lupus-prone mice WT or MRL/*lpr* mice were retro-orbitally infected with *S. aureus* and treated with vehicle, HCQ (40 mg/kg/every other day), and/or anti-IFNAR1 antibody (10 mg/kg/every other day). (A) Mouse survival was monitored daily post-infection. (B–E) At 6 days post-infection, (B) bacterial burdens in the heart, liver, and kidney were quantified by dilution spot plating and neutrophils from the tissues analyzed for (C) LDHB protein expression relative to isotype control, (D) suicidal NETosis, and (E) vital NET release by flow cytometry. Data represent (A) the mean survival of mice, and (B–E) each point represents a single mouse. (A) Log rank (Mantel-Cox) test and (B–E) two-way ANOVA with Tukey multiple-comparisons test; error bars reflect standard error (*p ≤ 0.05, **p ≤ 0.01, ***p ≤ 0.001, ****p ≤ 0.0001; ns, not significant).

IFNα plays a critical role in driving SLE pathology, and similarly, MRL/*lpr* mice have increased serum IFNα (Figure S4A). Thus, high circulating IFNα may exacerbate vital NET release during infection, impairing the transition to mitochondrial ROS-driven suicidal NETosis. To test this, WT and MRL/*lpr* mice were systemically infected with *S. aureus* by retro-orbital inoculation and treated with either vehicle, HCQ, an anti-IFNAR1 antibody, or both. MRL/*lpr* mice had elevated autoantibody titers and mild glomerulonephritis (proteinuria score ≤2) at the time of infection (Figures S4B and S4C). Both WT and MRL/*lpr* mice treated with anti-IFNAR1 were highly susceptible to *S. aureus* bacteremia. Dual treatment with HCQ and anti-IFNAR1 in MRL/*lpr* mice did not significantly improve survival (Figure 7A) despite similar weight loss across treatment groups (Figure S4D). However, bacterial burdens in the heart and liver were significantly decreased with a strong trend observed in the kidney in dual-treated MRL/*lpr* mice relative to vehicle controls (Figure 7B). The decrease in bacterial burdens coincided with restoration of LDHB expression (Figures 7C and S4E), suicidal NETosis (Figures 7D and S4F), and normalization of vital NET release (Figures 7E and S4G). These results demonstrate that combined HCQ and IFNAR1 blockade restores mitochondrial function and rebalances NET responses in lupus-prone mice, improving bacterial clearance. However, the modest survival benefit despite improved bacterial control suggests that neutrophil dysfunction is only one facet of infection susceptibility in SLE. These findings underscore the multifactorial nature of immune dysregulation in lupus and highlight the need to address broader inflammatory and tissue-damaging pathways in this setting.

### HCQ and anifrolumab therapies restore regulation of NET release by SLE neutrophils

**Figure 8.**
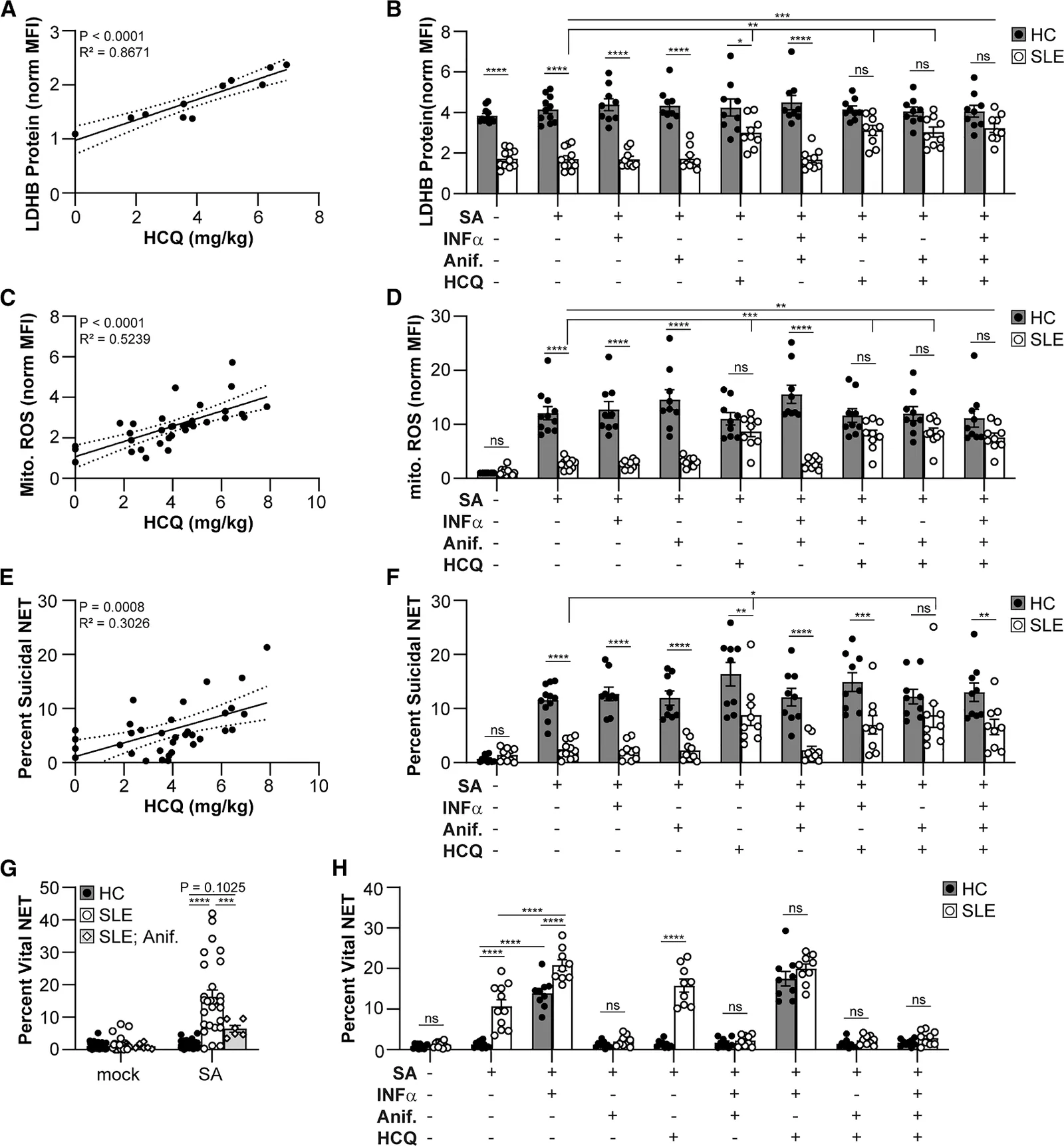
HCQ and anifrolumab therapies restore NET subtype regulation in SLE neutrophils Neutrophils were isolated from HC or SLE patients. (A and B) LDHB protein expression relative to isotype control in (A) unstimulated neutrophils or (B) neutrophils stimulated with *S. aureus* was quantified by flow cytometry. (A) LDHB expression was graphed relative to current dosage of HCQ. (B) Neutrophils were treated with IFNα, HCQ, and/or anifrolumab (anif.) and stimulated with *S. aureus* for 3 h. (C–H) Neutrophils were stimulated with *S. aureus*, and (C and D) mitochondrial ROS, (E and F) suicidal NETosis, and (G and H) vital NET release were quantified by flow cytometry. (C and E) Mitochondrial ROS and suicidal NETosis were graphed relative to current dosage of HCQ. (G) Vital NET release was quantified in patients not receiving or receiving anifrolumab treatment. (D, F, and H) Neutrophils were treated with IFNα, HCQ, and/or anifrolumab and stimulated with *S. aureus* for 3 h. Each point represents neutrophils isolated from a single donor. Data were quantified by (A, C, and E) linear regression or (B, D, and F–H) one-way ANOVA with Tukey multiple-comparisons test; error bars reflect standard error (*p ≤ 0.05, **p ≤ 0.01, ***p ≤ 0.001, ****p ≤ 0.0001; ns, not significant). (C, E, and G) Includes data from this study and prior studies.^34,37^

MRL/*lpr* mice are genetically identical and housed in controlled conditions, whereas SLE in patients arises from diverse genetic and environmental influences. To determine whether the mechanisms identified in lupus-prone mice extend to human disease, we analyzed neutrophils from individuals with SLE (Tables S1 and S2). Neutrophils were isolated from healthy controls (HCs) and SLE patients, and LDHB protein expression was quantified by flow cytometry. SLE neutrophils from patients receiving higher doses of HCQ, normalized to body weight, expressed significantly more LDHB compared to those on lower doses, though levels remained reduced relative to HC neutrophils (Figures 8A and 8B; Figure S5A). *Ex vivo* treatment of SLE neutrophils with *S. aureus* and HCQ increased LDHB expression to levels comparable to HC controls. To determine whether HCQ and anifrolumab similarly corrected the neutrophil response programs identified in the mouse studies, we next assessed mitochondrial ROS production (Figures 8C and 8D; Figure S5B) and suicidal NETosis (Figures 8E and 8F) in response to *S. aureus*. We analyzed responses both in freshly isolated neutrophils and by retrospectively correlating HCQ dosing with prior data.^34,37^ SLE neutrophils from patients on higher doses of HCQ produced more mitochondrial ROS and underwent greater suicidal NETosis following *S. aureus* challenge (Figures 8C–8E; Figure S5C). In addition, *ex vivo* HCQ treatment restored both responses to levels seen in HC neutrophils (Figures 8D–8F; Figure S5C). These findings indicate that HCQ dose-dependently restores LDHB expression and mitochondrial programming, thereby rescuing the capacity for suicidal NETosis in SLE neutrophils.

We next assessed chronic vital NET release in response to *S. aureus*. Neutrophils from SLE patients receiving anifrolumab exhibited significantly less vital NET release compared to untreated patients (Figures 8G and S5D). Furthermore, anifrolumab suppressed vital NET formation to levels observed in HC neutrophils, while exogenous IFNα augmented vital NET release in HC neutrophils to levels resembling SLE (Figures 8H and S5D). Together, these findings demonstrate that the aberrant NET program observed in lupus-prone mice is recapitulated in human SLE neutrophils and can be pharmacologically reversed. Although bacterial survival was not directly measured in these human treatment experiments, HCQ restored LDHB expression, mitochondrial function, and suicidal NETosis, while anifrolumab suppressed IFNα-driven vital NET release, indicating correction of the neutrophil response programs associated with impaired antibacterial defense.

## DISCUSSION

Neutrophils are essential for early antibacterial defense, yet in SLE, their responses become paradoxically maladaptive. Excessive NET formation fuels autoimmunity,^27–29^ while susceptibility to infections like *S*. *aureus* remains high.^46–49^ This contradiction suggests that not all NETs are equal in function. Here, we reveal that chronic innate immune activation in lupus reshapes NET output from bactericidal suicidal NETosis to a sustained, less bactericidal vital NET program, undermining host defense despite increased NET burden. We identify two linked processes that reshape NET responses in lupus. First, sustained endosomal TLR7/9 engagement represses LDHB, a mitochondrial enzyme required for lactate sensing and ROS production during bacterial infection,^34^ thereby impairing the suicidal NET pathway. Second, type I interferon signaling sustains a vital NET program that is less bactericidal and remains exaggerated in lupus-prone neutrophils. Together, these changes shift NET output away from an effective antibacterial suicidal response and toward a less protective vital NET state.

We further elucidate the upstream regulation of vital NET release. Staphylococcal Hla facilitates calcium influx and degranulation, initiating rapid IFNα secretion and vital NET formation. In healthy neutrophils, this occurs via a tightly regulated process that depends on intact calcium signaling. This is consistent with earlier work demonstrating that *S. aureus* triggers rapid, vesicle-mediated NET release via non-lytic, oxidant-independent pathways, particularly in response to PVL and related pore-forming toxins.^3^ More recently, type I interferon was shown to enable neutrophils to extrude NETs while maintaining membrane integrity in *Mycobacterium tuberculosis* granulomas.^36^ These findings align with our observation that IFNα signaling amplifies vital NET release and suggest that this pathway, while beneficial in some contexts, may become pathologically unrestrained in SLE. In lupus-prone neutrophils, IFNα release becomes decoupled from calcium dependence, enabling constitutive IFNAR signaling that sustains vital NET formation. Our findings reconcile previously conflicting reports on Hla’s effects on calcium flux and neutrophil activation^60–65^ and position vital NETosis as a context-dependent, IFNα-amplified program that contributes to defective host defense in lupus.

This NET subtype imbalance has functional consequences. Vital NETs lack the antimicrobial protein content and killing capacity of suicidal NETs yet are produced in greater abundance. In lupus-prone neutrophils, this imbalance not only fails to control *S. aureus* but also suppresses the more effective suicidal program, compounding infection risk. Both vital and suicidal NETs from MRL/*lpr* neutrophils contained elevated levels of S100A9, consistent with the increased calprotectin expression we previously reported in lupus neutrophils.^37^ Although calprotectin contributes to antibacterial defense,^69,70^ our findings demonstrate that its enrichment alone is insufficient to compensate for the loss of effective suicidal NETosis. These results align with recent evidence that *S. aureus* PVL triggers vital NET formation, yielding NETs with reduced NE, cathelicidin, and proteinase 3 content and poor bactericidal activity.^71^ It is also important to note that our H3Cit measurements were made on released NET material after prolonged stimulation and therefore likely reflect the final H3Cit content of extracellular NETs rather than early intracellular histone citrullination kinetics. This likely explains why our endpoint differs from that in prior studies in which A23187 induced more rapid cellular H3Cit accumulation at earlier time points.^59^ More broadly, the skew toward vital NET release exacerbates functional defects, impairing bacterial clearance despite continued NET deposition. These findings resolve a long-standing paradox in lupus pathogenesis: NET abundance is not protective when the dominant subtype lacks bactericidal potency.

Importantly, this dysregulated NET program is therapeutically reversible. In lupus-prone mice, HCQ restores LDHB expression, mitochondrial ROS, and suicidal NETosis by blocking endosomal TLR7/9 signaling. Notably, although the clinical benefits of HCQ in SLE typically emerge over a longer time frame at the level of systemic disease, the short HCQ treatment used in our mouse studies was designed to assess rapid neutrophil-intrinsic reprogramming. Given the short half-life and rapid turnover of neutrophils, such rapid changes in neutrophil phenotype are biologically plausible in this experimental context. IFNAR blockade with anti-IFNAR1 antibody disrupts IFNα-driven vital NET release. Combined therapy rebalances NET subtypes and reduces bacterial burden *in vivo.* In human SLE neutrophils, defects in LDHB and NET programming mirror those in mice and are corrected by HCQ or anifrolumab, providing mechanistic rationale for these therapies and their benefits to innate immunity.

However, rebalancing NET responses alone was insufficient to improve survival in septic lupus-prone mice, highlighting the multifactorial nature of infection vulnerability in SLE. Factors such as impaired macrophage function,^25,26,72–75^ vascular pathology,^14–20^ and complement deficiencies^76^ likely contribute to poor outcomes. Moreover, it remains to be seen whether similar NET imbalances impair responses to other pathogens, including viruses and fungi, particularly given the critical role of IFN signaling in antiviral defense.

Our findings underscore several broader implications. First, NET subtype identity is functionally decisive, not merely morphological. Suicidal NETosis is linked to robust antimicrobial content and cell death, whereas vital NETs are non-lytic, less antimicrobial, and suppress more effective NET programs. Second, LDHB emerges as a central metabolic node linking innate immune signaling to mitochondrial fitness and effector function, one that may be dysregulated across multiple cell types and diseases marked by chronic inflammation.^77–80^ Finally, NET subtype composition may represent a tractable biomarker of neutrophil health and treatment response in SLE and other inflammatory diseases.

In summary, this study shows that TLR7/9 and type I interferon signaling reprogram neutrophil NET responses in lupus, shifting antibacterial defense away from bactericidal suicidal NETosis and toward a less protective vital NET state. By defining how chronic inflammation impairs antibacterial immunity through metabolic and signaling reprogramming, we reveal an actionable mechanism that reconciles key paradoxes in SLE pathogenesis and lays the groundwork for targeting innate dysfunction in autoimmune disease.

### Limitations of the study

This study has several limitations. MRL/*lpr* mice model key features of SLE but do not capture the full heterogeneity of human disease, and the human SLE cohort size limited our ability to stratify responses by disease activity, medication exposure, or infection history. In addition, the 16-h priming assay should be interpreted as a defined inflammatory conditioning model rather than an unstimulated baseline state, since neutrophils required IFNγ and TLR agonist stimulation to maintain sufficient viability. Finally, vital and suicidal NET responses were measured at time points selected to capture their characteristic kinetics, so endpoint timing and detection thresholds may influence the relative abundance of each NET population. Despite these caveats, the mouse and human data converge on a model in which SLE-associated signaling impairs LDHB-associated suicidal NETosis and promotes less bactericidal vital NET release.

## RESOURCE AVAILABILITY

### Lead contact

Any requests for further information regarding reagents, methods, etc. should be directed to the corresponding author, Dr. Andrew Monteith (amonteit@utk.edu).

### Materials availability

All unique reagents are available upon request to the corresponding author.

### Data and code availability

All data reported in this study are available from the lead contact upon reasonable request. This paper does not report original code.

## STAR★METHODS

### EXPERIMENTAL MODEL AND STUDY PARTICIPANT DETAILS

#### Ethics statement

Protocols used on mice in this study were reviewed and approved by the University of Tennessee Institutional Animal Care and Use Committee (protocol number 2945–1222) in accordance with the Public Health Service Policy on the Human Care and Use of Laboratory Animals under the Unites States of America National Institutes of Health (NIH) Office of Laboratory Animal Welfare (OLAW). Written informed consent was obtained from all donors, and experimentation performed on these samples was approved by the University of Tennessee Knoxville Institutional Review Board (IRB-22–07231-XP) and UT Graduate School of Medicine in Knoxville (5034).

#### Bacteria

All *S. aureus* experiments used the bacterial strain USA300 (LAC). The Δ*nuc* mutant^1^ was from the Horswill lab. The ΔPVL strain was from the Nebraska Transposon Mutant Library^82^ and the Δ*hla* mutant^83^ was from Megan Kiedrowski. Bacteria were maintained as a −80°C glycerol stock, and *S. aureus* was streaked onto tryptic soy agar (TSA; 2% agar) plates to grow overnight at 37°C. Single colonies were transferred to tryptic soy broth (TSB) and liquid cultures were grown overnight at 37°C with 250 rpm of shaking. For all *in vitro* experiments, overnight cultures were diluted 1:50 into non–heat-inactivated FBS before coculture with neutrophils.

#### Mice

C57BL/6J and MRL/MpJ-*Fas*^*lpr*^/J mice were purchased from the Jackson Laboratory (JAX mice stock no. 000664 and no. 000485). A C57BL/6J breeding colony was subsequently maintained in an accredited animal facility. Mice were housed two to five mice per cage with an independently ventilated air supply and food and water were provided ab libitum. Cages were changed on a weekly basis. For hydroxychloroquine (HCQ) treatments, mice were injected intraperitoneally at a concentration of 40 mg/kg every 24 h for 2 days prior to neutrophil isolation. Mock treatment was the diluent (DPBS). All experiments were conducted in accordance with protocols approved by the University of Tennessee Institutional Animal Care and Use Committee.

#### Human samples

Peripheral blood was collected from either healthy human donors or patients with systemic lupus erythematosus (SLE). All participants gave their informed consent, and all procedures involving human blood were approved by the University of Tennessee Knoxville Institutional Review Board (IRB-22–07231-XP) and UT Graduate School of Medicine in Knoxville (5034). Age, sex, and ethnicity was recorded for each donor and the lupus criteria, SLICC, and SLEDAI-2K scores were determined upon donation. Information provided in Tables S1–S3.

### METHOD DETAILS

#### Human neutrophil isolation

Neutrophils were isolated from the peripheral blood of donors using density centrifugation where neutrophils were isolated at the interface between layers of Histopaque 1119 and Histopaque 1077. Serum was pulled off the top of the blood prior to density centrifugation each experiment for opsonization of bacteria. Blood was diluted with DPBS then layered on top of 2–3 Histopaque stacks. Isolated neutrophils were washed with DMEM and then incubated on ice for 45 min in DMEM with 10% FBS (D10 media) before lysing red blood cells with 1–2mL 1X RBC lysis buffer. The isolated neutrophils rested 1 h at 37°C, 5% CO_2_ before experiments. The isolated cells were 90–95% neutrophils (CD15^+^CD16^+^), as verified by flow cytometry.

#### Murine neutrophil isolation

Single-cell suspensions of bone marrow were extracted from the tibias and femurs of female mice aged 6–10 weeks and layered on top of a Histopaque stack. Following density centrifugation, the neutrophils were isolated at the interface of Histopaque 1077 and Histopaque 1119. The cells were washed with DMEM and resuspended in D10 media. The neutrophils were rested on ice for 1 h, plated, and allowed to rest for 1 h at 37°C, 5% CO_2_ before experiments. Alternatively, neutrophils were cultured with IFNγ and TLR agonists (50 ng/mL) for 16 h at 37°C, 5% CO_2_ before conducting experiments. The isolated cells were 85–95% neutrophils (CD11b^+^Ly6G^+^), as verified by flow cytometry.

#### Apoptotic debris

Following euthanasia, the thymus was removed from a mouse less than 15 weeks of age, grinded through a 70 μm cell strainer and rinsed with DPBS. Cells were irradiated (6 gray), placed in a 6 cm Petri dish, and incubated at 37°C, 5% CO_2_ overnight. The next day the sample was transferred to a 15 mL conical tube and pelleted at 350 × g for 5 min. Supernatant containing apoptotic debris was transferred to a fresh tube and added to serum to stimulate neutrophils.

#### Murine bacteremia models

*S. aureus* was streaked from frozen stocks on TSA. Overnight cultures of *S. aureus* were grown in TSB and then subcultured 1:100 in 5 mL of TSB. *S. aureus* was grown for 3 h, washed, and resuspended in ice-cold DPBS. Bacteria was resuspended at 2×10^8^ CFU/mL for C57BL/6 or 4×10^8^ CFU/mL for MRL/*lpr* to account for the differences in size between the two mice. All infections used 10- to 12-week-old female mice, which were anesthetized using inhalation of isoflurane followed by inoculation of the retro-orbital sinus with 100 μL of bacterial slurry or mock treated with 100 μL DPBS. A lubricant eye ointment was applied after each injection and mice were monitored until they regained consciousness. Mice were monitored daily before humane euthanasia by inhalation of CO_2_.

For anti-IFNAR (anifrolumab) and HCQ treatments, mice were injected intraperitoneally every other day beginning the day after infection. Anti-IFNAR was administered at a concentration of 10 mg/kg and HCQ at 40 mg/kg. Mock treatments were the respective diluent.

One day prior to infection, murine levels of proteinuria and serum autoantibody were quantified. Urine protein was assessed according to Uristix 4 (Siemens) instruction. Blood was collected by tail nick and serum levels of IgG reactive to dsDNA was quantified by ELISA.

#### Organ Harvest

Following euthanasia, the heart, liver, and kidney were removed, weighed and placed into tubes containing DPBS on ice. Blood was collected from cardiac sticks for IFNα ELISA. Single cell suspensions were created by grinding organs through 70 μm cell strainers and rinsing in DPBS. Aliquots of 200 μL of the single cell suspension were reserved for serial dilution spot plating before centrifugation to determine bacterial load. Cells were pelleted and the pellets resuspended with 1X RBC lysis buffer per manufacturer’s instructions. Cells were strained through a 70 μm cell strainer, pelleted by centrifugation, washed in DPBS, and resuspended in FACs media (DPBS with 2% heat inactivated FBS and 0.02% NaAz) and plated for flow cytometry.

#### Bacterial burdens from infections

Samples were aliquoted into the top row of a 96 well plate and then serially diluted in DPBS using 10-fold increments down the plate. 5 μL of each dilution was then spotted onto TSA plates and incubated for 12–16 h in a static 37°C incubator. Plates were removed from the incubator and CFU counted.

#### Flow cytometry

An Attune NXT flow cytometer was used to run all flow cytometry assays. All data was analyzed using FlowJo software. For all flow cytometry data, cells were gated side scatter height (SSC-H) by side scatter area (SSC-A) followed by forward scatter height (FSC-H) by forward scatter area (FSC-A) to remove doublet populations. The singlet population was gated SSC-H by FSC-H to isolate the granulocyte populations. The resulting cell population was then assessed for assay-specific fluorescent markers as described below.

##### Exogenous treatments

Neutrophils were pre-treated with HCQ (30 pM) 30 min prior to stimulation and EDTA (0.5mM) 5 min prior to stimulation. Neutrophils were stimulated with bacteria at an MOI = 10. Cl-amidine (34 μg/mL), IFNα (200 units/well), α-IFNAR (10 μg/mL), Pam2CSK4 (1 μg/mL), Pam3CSK4 (1 μg/mL), Poly I:C (20 μg/mL), LPS (1 μg/mL), imiquimod (2 μg/mL), CpG ODN (45 nM), sodium lactate (1 mM), and/or Hla protein (1 μg/mL) were added concurrently with bacteria. A23187 (5 μM) and PMA (50 nM) were added to trigger vital NET release or suicidal NETosis. Alternatively, A23187 or PMA were added 30 min prior subsequent simulations or bacteria.

##### Mitochondrial ROS flow

Following plating, neutrophils were stained with mitoTracker (500 nM) deep red and mitoSOX red (5 μM) dyes for 20 min, washed, and fresh D10 media added to the neutrophils. Neutrophils were stimulated for 3 h. Neutrophils were stained with Ghost Dye Violet 540 to monitor membrane integrity 20 min prior to fixation. Neutrophils were fixed with room temperature 4% PFA and placed on ice for 20 min. Neutrophils were pelleted and blocked in FACS media (mouse = Fc Shield; human = Human TruStain FcX) on ice for 20 min. Neutrophils were pelleted, and resuspended in FACS media containing fluorescent antibodies (mouse = α-CD11b, α-Ly6G; human = α-CD16, α-CD15) for 20 min on ice. Neutrophils were washed and resuspended in FACS media for analysis. Cells were gated for live cells (Ghost^−^) and then neutrophils (CD11b^+^Ly6G^+^ or CD15^+^CD16^+^). mitoSOX and mitoTracker mean fluorescence intensity (MFI) were normalized to untreated or mock WT MFI.

##### NETosis flow

NETosis flow was conducted as previously described.^7^ Neutrophils were stimulated for 30 min or 3 h and Ghost Dye Violet 540 and SYTOX blue (500 nM) were added to the culture 20 min prior to fixation to monitor membrane integrity and extracellular DNA, respectively. Neutrophils were fixed with room temperature, 4% PFA for 20 min on ice and subsequently blocked in FACS media (mouse = Fc Shield, human = Human TruStain FcX) on ice for 20 min. Neutrophils were pelleted and resuspended in FACS media containing fluorescent antibodies (mouse = α-CD11b, α-Ly6G; human = α-CD16, α-CD15) and biotinylated α-myeloperoxidase and rabbit α-H3Citruline for 20 min on ice. Neutrophils were pelleted and resuspended in FACS media containing fluorescently-labeled streptavidin and α-rabbit antibodies on ice for 20 min. Neutrophils were washed and resuspended in FACS media for analysis. Cells were gated for neutrophils (CD11b^+^Ly6G^+^ or CD15^+^CD16^+^) and cells positive for extracellular dsDNA (SYTOX^+^), MPO, and H3Cit were defined as having NET release. Neutrophils releasing NETs that have permeabilized membranes (Ghost^+^) were defined as having undergone suicidal NETosis, while Neutrophils releasing NETs that do not have permeabilized membranes (Ghost^−^) were defined as having undergone vital NET release. The percentage of neutrophils undergoing NETosis was quantified relative to the total number of neutrophils.

##### LDH protein flow

To quantify intracellular LDH abundance, neutrophils were stained with Ghost Dye Violet 540 20 min prior to fixation, fixed with room temperature 4% PFA for 20 min on ice, blocked in FACS media (mouse = Fc Shield; human = Human TruStain FcX) on ice for 20 min, and stained in FACS media containing fluorescent antibodies (mouse = α-CD11b, α-Ly6G; human = α-CD16, α-CD15) on ice. Neutrophils were then stained with a rabbit α-LDHA or -LDHB antibody or isotype control in Saponin Permeabilization Buffer for 20 min on ice, and stained with a fluorescent α-rabbit antibody in Saponin Permeabilization Buffer for 20 min on ice. Neutrophils were washed and resuspended in FACS media for analysis. LDH MFI was normalized to an isotype control.

##### Degranulation flow

Neutrophils were stimulated for 30 min and stained with Ghost Dye Violet 540 20 min prior to fixation, fixed with room temperature 4% PFA for 20 min on ice, blocked in FACS media (Fc Shield) on ice for 20 min, and stained with antibodies recognizing appropriate neutrophil surface markers (murine: CD11b, Ly6G; human: CD15, CD16), anti-CD63 (primary), and anti-CD35 (secretory) or appropriate isotype controls in FACS media for 20 min on ice. Cells were washed following staining and resuspended in FACS media for analysis. Cells were gated for live cells (Live/Dead-negative) and then neutrophils (CD11b- and Ly6G-positive). The median fluorescence intensity (MFI) was quantified for surface CD63 and CD35 relative to staining with an isotype control antibody.

##### Membrane Polarization flow

After an hour rest, neutrophils were stained with 250 nM DiBAC4(3) for 8 min at 37°C then stimulated for ten minutes and run on the flow cytometer. The median fluorescence intensity (MFI) was quantified for DiBAC4(3) and normalized to unstimulated neutrophils.

#### Confocal microscopy

NET Microscopy Neutrophils were stained with CellMask deep red (5 mg/mL) and incubated at 37°C for 30 min. Cells were washed and 4×10^5^ neutrophils were plated in 150 μL of D10 on a poly-L-Lysine coated 8 Well Chambered Cover Glass. After 1 h incubation at 37°C, 5% CO2 cells were stimulated with Baclight Red-stained strains of *S. aureus* for indicated times. Hoescht 33342 (10 mM) was added during the last 30 min of incubation. Following stimulation the media was aspirated and neutrophils fixed with 4% PFA for 20 min ProLong Gold Antifade reagent was then applied. For HCQ (30pM) treatment, neutrophils were pretreated for 30 min prior to stimulation with *S. aureus*. NETs were imaged on a ZEISS LSM 900 with Airyscan. 2 confocal microscope. Images were analyzed using ImageJ software.

#### Anti-dsDNA IgG ELISA

Vinyl 96-well plates were coated with dsDNA (10μg/mL) and incubated overnight at 4°C. The wells were washed before and after blocking and samples/standards were added before incubating overnight at 4°C. Captured antibodies were detected with anti-mouse IgG-alkaline phosphatase, A standard curve was generated using α-dsDNA and antibody. Absorbance at 405 nm for each well was quantified using a BioTek Cytation plate reader. Samples and standards were read in duplicate and DNA abundance was quantified relative to the standard.

#### IFNα ELISA

*In-vitro* supernatant samples and serum samples were diluted 1:3 and 1:10 respectively in assay dilution buffer. Total IFNα protein was quantified using Mouse IFNα ProQuantum Immunoassay Kit following manufacturer instructions. Plates were ran on a Quantstudio 3 (Applied Biosystems).

#### Mitochondrial protein immunoblot

Murine neutrophils were pooled from 2 mice and mitochondria were isolated from the cytosol (Thermo Scientific, 89874) per manufacturer instructions. Mitochondrial fractions were lysed using 1X RIPA containing protease inhibitors. Mitochondrial lysates were heated for 5 min at 100°C in the presence of Laemmli loading buffer containing β-mercaptoethanol. A volume of 25 μL/well was loaded onto a 15% polyacrylamide gel, followed by electrophoresis. Gels were transferred onto nitrocellulose using a Trans-Blot Turbo and blocked at 4°C with Intercept (TBS) blocking buffer for 4 h before an overnight incubation in either primary LDHB or SOD2 diluted at 1:1,000 in TBST with 1% milk. Secondary antibody incubations were done for 4 h with 1:10,000 goat anti-rabbit IgG HRP before developing with a Chemiluminescent Substrate kit (Thermo Scientific, 34580) per manufacturer instructions. Blots were then imaged on an iBright 1500 and densitometry quantified by ImageJ software.

#### Fluorescence plate reader assays

##### Calcium

Murine neutrophils were isolated and resuspended in clear D10 and stained with Fluo-4 calcium dye (1:2,000) for 40 min and Hoechst dye (1:1000) for 20 min. The cells were washed and plated (250,000/well) in clear D10 media in a black-wall, clear bottom, 96-well plate that was Poly-L-Lysine coated. After an hour rest at 37°C, 5% CO_2_, fluorescence intensity of total DNA (Hoescht 33342) and calcium were read on a Biotek Cytation plate reader. Neutrophils were stimulated with bacteria at an MOI of 10 and Hoechst 33342 and Fluo-4 fluorescence was read every 5 min for 1 h.

##### Characterization of A23187 and PMA induced NETs

Murine neutrophils were isolated and plated (500,000/well) on a Poly-L-Lysine coated, black wall, clear bottom plate. A23187 (5 μM) and PMA (50 nM) were added to the neutrophils and the plate was cultured for 16 h at 37°C, 5% CO_2_. Wells were stained with SYTOX blue (500 nM) to quantify total DNA and antibodies specific to MPO, NE, and H3Cit with appropriate fluorescent secondary antibodies to quantify protein abundance.

##### NE activity

Neutrophils were stimulated for 30 min and supernatants were taken from isolated and 15 μL transferred to the assay plate provided. NE activity was quantified per manufacturer’s instructions and read on a plate reader at an excitation/emission wavelength of 485/525 nm. Activity of samples were normalized to the standard curve provided by the kit.

#### Dilution spot plate assays

##### Neutrophil killing assay

Neutrophils were seeded at 2×10^5^ cells/well in a 96-well plate and were allowed to rest for 30 min in a 37°C, 5% CO_2_ incubator. Neutrophils were challenged with *S. aureus* opsonized in 1:1 DMEM:non-heat inactivated FBS at a MOI = 0.5. After 4 h, samples were serially diluted and spot-plated onto TSA. Percent growth was quantified relative to bacteria grown in the absence of neutrophils.

##### NET killing assay

Murine neutrophils were isolated and plated (500,000/well) on a Poly-L-Lysine coated, black wall, clear bottom plate. A23187 (5 μM) and PMA (50 nM) were added to the neutrophils and the plate was cultured for 16 h at 37°C, 5% CO_2_. Neutrophils were challenged with *S. aureus* at a MOI = 0.2. After 4 h, samples were serially diluted and spot-plated onto TSA. Percent growth was quantified relative to bacteria grown in the absence of neutrophils.

#### Quantitative PCR

RNA was extracted from murine neutrophils using the Qiagen RNeasy mini kit following an enzymatic digestion with lysis buffer supplemented with lysozyme (2.88 mg/mL), mutanolysin (2.4 U/μL), proteinase K (3.85 mg/mL), and EDTA (19.2 mM). RNA was further purified by performing a second DNAse treatment with Turbo DNAse kit following the manufacturer’s protocol. RNA was quantified using a qubit RNA HS kit. 318 ng of RNA was then reverse transcribed to cDNA using the SuperScript III First-Strand Synthesis Kit. Quantitative PCR (qPCR) was performed using TaqMan assays to quantify transcript levels of *Ldha* (Mm01612132_g1 FAM) and *Ldhb* (Mm01267402_m1). Assays were prepared in triplicate with the TaqMan Gene-Expression Master Mix, and the plates were run on the QuantStudio 3 Real-Time PCR System (Applied Biosystems) following manufacturer instructions. Relative transcript abundance was calculated using the 2−ΔΔCt method, normalizing each target’s cycle threshold (Ct) to the endogenous control gene, *Actb* (Mm02619580_g1 VIC).

#### Quantification statistical analysis

Specific statistical details for each experiment are provided in the corresponding figure legend, including the statistical test used, exact value of n, what n represents, definition of center, and dispersion and precision measures. Error bars for all experiments represent standard error of the mean. A minimum of three experimental replicates were performed for each assay and the specific number of replicates is noted in the corresponding figure and figure legend. Statistical analyses were performed using Prism 6 software (GraphPad). Significance is indicated on the graphs as follows: *, *p* ≤ 0.05; **, *p* ≤ 0.01; ***, *p* ≤ 0.001; ****, *p* ≤ 0.0001; ns, not significant.

## Supplementary Material

1

SUPPLEMENTAL INFORMATION

Supplemental information can be found online at https://doi.org/10.1016/j.cpblue.2026.100080.

## Figures and Tables

**KEY RESOURCES TABLE:** 

REAGENT or RESOURCE	SOURCE	IDENTIFIER
Experimental models: Organisms/strains		
C57BL/6J	Jackson Labs	000664; RRID: IMSR_JAX:000664
MRL/*lpr* (MRL/MpJ-*Fas^lpr^*/J)	Jackson Labs	000485; RRID: IMSR_JAX:000485
TLR2^−/−^ (B6.129-*Tlr2tm1Kir*/J)	Jackson Labs	004650; RRID: IMSR_JAX:004650
TLR4^−/−^ (B6(Cg)-Tlr4tm1.2Karp/J)	Jackson Labs	029015; RRID: IMSR_JAX:029015
TLR7^−/−^ (B6.129S1-Tlr7tm1Flv/J)	Jackson Labs	008380; RRID: IMSR_JAX:008380
TLR9^−/−^ (C57BL/6-Tlr9em1.1Ldm/J)	Jackson Labs	034449; RRID: IMSR_JAX:034449
IFNAR^−/−^ (B6(Cg)-*Ifnar1tm1.2Ees*/J)	Jackson Labs	028288; RRID: IMSR_JAX:028288
Biological samples		
Human blood samples	Donors at UTK or UTMC	N/A
Bacterial and virus strains		
*S. aureus* USA300 LAC WT	Gift from Eric Skaar	N/A
*S. aureus* Δ*nuc*	Gift from Horswill	Kiedrowski et al.^81^
*S. aureus* Δ*pvl*	NTML	Fey et al.^82^
*S. aureus* Δ*hla*	Gift from Megan Kiedrowski	Olson et al.^83^
S. aureus Δ*hla* + *hla*	Gift from Megan Kiedrowski	Olson et al.^83^
Oligonucleotides		
LDHA	Thermo Scientific	Mm01612132_g1, FAM
LDHB	Thermo Scientific	Mm01267402_m1, FAM
ACTB	Thermo Scientific	Mm02619580_g1, VIC
Antibodies		
α-dsDNA	Abcam	AB27156; RRID: AB_470907
h3cit	Abcam	AB5103; RRID: AB_304752
IgG alkaline phosphatase	Abcam	AB98724; RRID: AB_10671655
α-MPO	Abcam	AB90810; RRID: AB_2146325
α-NE	Abcam	AB68672; RRID: AB_1658868
mouse α-IFNAR	Biolegend	127324; RRID: AB_2629522
α-rabbit A488	Biolegend	406416; RRID: AB_2563203
α-rabbit 647	Biolegend	406414; RRID: AB_2563202
streptavidin A488	Biolegend	405235
α-CD15	Biolegend	323050; RRID: AB_2750192
α-CD16	Biolegend	302016; RRID: AB_314216
α-CD63	Biolegend	143903; RRID: AB_11203532
α-CD21/35	Biolegend	123409; RRID: AB_940411
PE isotype control	Biolegend	400508
α-CD11b	Biolegend	60-0112-U100
Human Trustain FcX	Biolegend	422302-BL
anti-Ly6G	Biolegend	127643; RRID: AB_2565971
anti-SOD2	Cell Signaling	D3X8F
anti-LDHB	Invitrogen	MA541126; RRID: AB_2898880
anti-LDHA	Invitrogen	PA523036; RRID: AB_11152155
Goat anti-rabbit IgG HRP	Promega	W4011; RRID: AB_430833
human α-IFNAR	Selleck	A2460
Fc Shield	Tonbo	70-0161-M001
α-S100A9	Cell Signaling	73425; RRID: AB_2799839
Critical commercial assays		
Chemiluminsecent substrate kit	Thermo Scientific	34580
Mitochondrial isolation kit	Thermo Scientific	89874
Mouse IFNa ProQuantum Immunoassay Kit	Invitrogen	A46736
Chemicals, peptides, and recombinant proteins		
DiBAC4(3)	Invitrogen	B438
MitoSOX red	Invitrogen	M36008
MitoTracker deep red	Invitrogen	M22426
SYTOX blue	Invitrogen	S34857
Fluo-4	Thermo Scientific	F14201
Hoechst 33342	Thermo Scientific	62249
Ghost Dye Violet 540	Tonbo	13-0879-T100
Laemmli loading buffer	BioRad	1610747
10x RBC lysis buffer	Biolegend	420302
mouse IFNα	PeproTech	315-05-20UG
PFA	Electron Microscopy Sciences	15710
EDTA	Fisher	BP2482100
DMEM	Gibco	11-965-118
DPBS	Cytiva	16750-102
FBS	Gibco	A52567-01 Lot 2635802RP
salmon sperm (dsDNA)	Invitrogen	15632011
CpG ODN	Invivogen	TLRL1826
human IFNα	PeproTech	300-02AA-250
mouse IFNγ	PeproTech	315-05-250UG
A23187	Sigma-Aldrich	AC328080050
Cl-Amidine	Sigma-Aldrich	506282-10MG
Histopaque 1077	Sigma-Aldrich	10771-6X100ML
Histopaque 1119	Sigma-Aldrich	11191-6X100ML
Hla protein	Sigma-Aldrich	H9395-.5MG
LPS	Sigma-Aldrich	L4391-10X1MG
PMA	Sigma-Aldrich	P8139-5MG
Poly-L-Lysine	Sigma-Aldrich	p4707
NaLactate	Sigma-Aldrich	71718
Imiquimod	Tocris	3700
Pam2CSK4	Tocris	4637
Pam3CSK4	Tocris	4633
Poly I:C	Tocris	4287
Software and algorithms		
Flow Jo	N/A	https://www.flowjo.com/solutions/flowjo/downloads
ImageJ	N/A	https://imagej.net/ij/download.html
Graph Pad Prism	N/A	https://www.graphpad.com/

## Highlights

- SLE neutrophils have reduced LDHB and impaired lactate-driven suicidal NETosis
- TLR7/9 and type I IFN signaling shift SLE neutrophils toward vital NET release
- Vital NETs are less bactericidal and limit effective antibacterial NET responses
- HCQ and IFNAR blockade rebalance SLE NET programs and improve bacterial clearance
