# Supplementary material for: TLR7/9 and type I interferon signaling reprogram neutrophil NET responses and impair antibacterial defense in systemic lupus erythematosus
Source: Cell Press Blue. Author manuscript; Available in PMC 2026 Sep 16. (PMC13576733; doi:10.1016/j.cpblue.2026.100080)
Supplement: Supplementary file 1 [file NIHMS2205050-supplement-1.pdf]

CPBLUE, Volume *1*

Supplemental information

TLR7/9 and type I interferon signaling reprogram neutrophil NET responses and impair antibacterial defense in systemic lupus erythematosus

Eden G. TenBarge, Ashley D. Wise, Morgan L. Hetzel, Helene A. Hoover, Helia Esfandiari, Bailey E. Holder, Eva Belevska, Ellie C. Mennen, Sarah R. McDaniel, Nicole M. Vaccaro, Courtney C. Lucca, Jonathan M. Williams, Jennifer Ferris, Timothy E. Sparer, Leslie J. Crofford, Jeffry D. Bieber, and Andrew J. Monteith

Supplemental Data

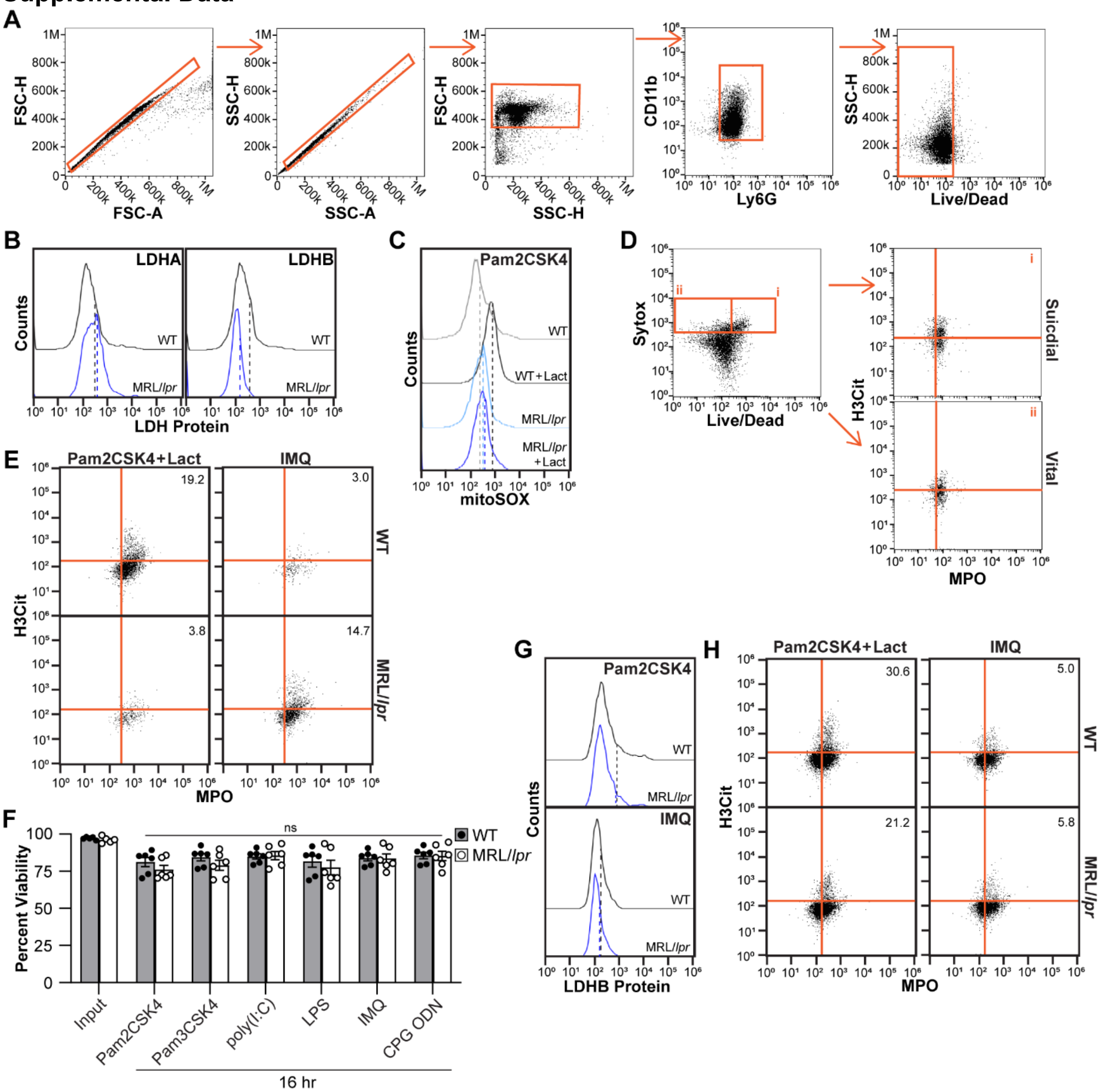

Supplemental Figure 1: TLR signals differentially shape LDHB expression and suicidal NETosis in WT and lupus-prone neutrophils. (**A**) Representative flow plots providing the gating scheme to identify live neutrophils. This gating proceeded assays quantifying intracellular LDHA/LDHB, mitochondrial ROS, membrane potential, and degranulation. (**B**) Neutrophils were isolated from WT or MRL/*lpr* and total LDHA or LDHB protein were quantified by flow cytometry. (**C-E**) WT or MRL/*lpr* neutrophils were treated with differing TLR agonists in the presence or absence of lactate (1 mM) and (*C*) mitochondrial ROS or (*E*) suicidal NETosis were quantified by flow cytometry. (*D*) Representative flow plots illustrating the gating strategy used to identify neutrophils undergoing suicidal NETosis (i) or vital NET release (ii). Cells were first gated as shown in panel A to identify neutrophils and then further gated to distinguish NET release programs. Neutrophils were identified by extracellular double-stranded DNA (Sytox Blue), MPO, and H3Cit staining, while membrane integrity was assessed with a fluorescent viability dye (Ghost dye 540). Neutrophils positive for extracellular dsDNA, MPO, and H3Cit with permeabilized

membranes were classified as undergoing suicidal NETosis, whereas neutrophils positive for extracellular dsDNA, MPO, and H3Cit with intact membranes were classified as undergoing vital NET release. (**F**-**H**) Neutrophils were stimulated with TLR agonist (50 ng/mL) and IFNγ (50 ng/mL) and cultured for 16 hours. (*F*) Viability of input or neutrophils following the 16 hour culture were quantified by flow cytometry. Neutrophils were cultured with *S. aureus* for 3 hours and (*G*) total LDHB protein or (*H*) suicidal NETosis were quantified by flow cytometry. (*F*) Two-way ANOVA with Tukey multiple comparisons test; error bars reflect standard error (ns = not significant). (*B-C, G*) Dashed line = MFI.

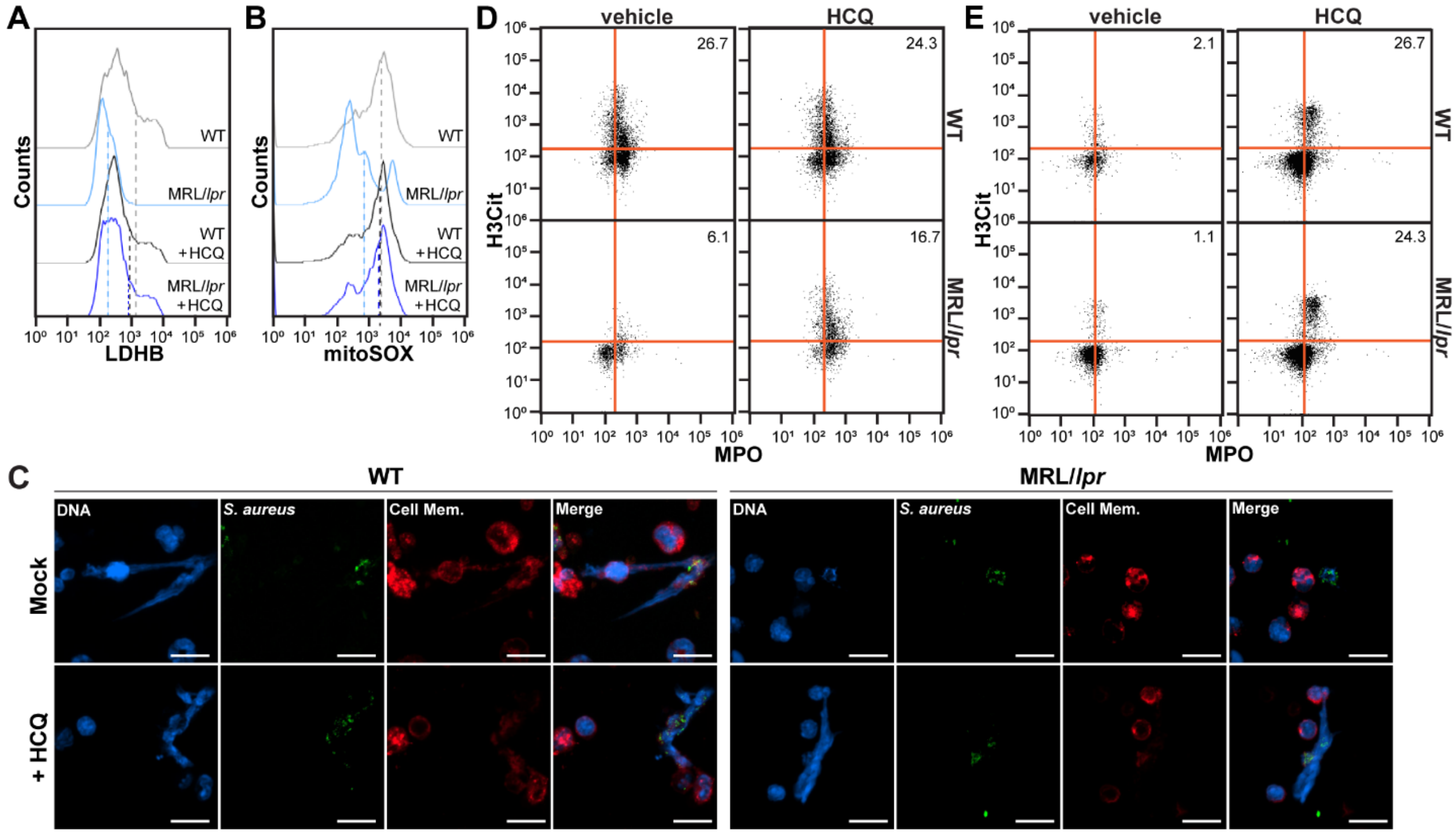

Supplemental Figure 2: HCQ restores mitochondrial ROS and suicidal NETosis but fails to prevent chronic vital NET release in MRL/*lpr* neutrophils. WT or MRL/*lpr* mice were mock treated or treated with HCQ (40 mg/kg) daily for 2 days. Neutrophils were isolated from the bone marrow, cultured with *S. aureus* for 3 hours, and (*A*) total LDHB protein, (*B*) mitochondrial ROS, neutrophils undergoing (*D*) suicidal NETosis or (*E*) vital NET release were quantified by flow cytometry. (*C*) Deposition of NETs were also quantified by confocal microscopy (bar = 10 µm). (*A*-*B*) Dashed line = MFI.

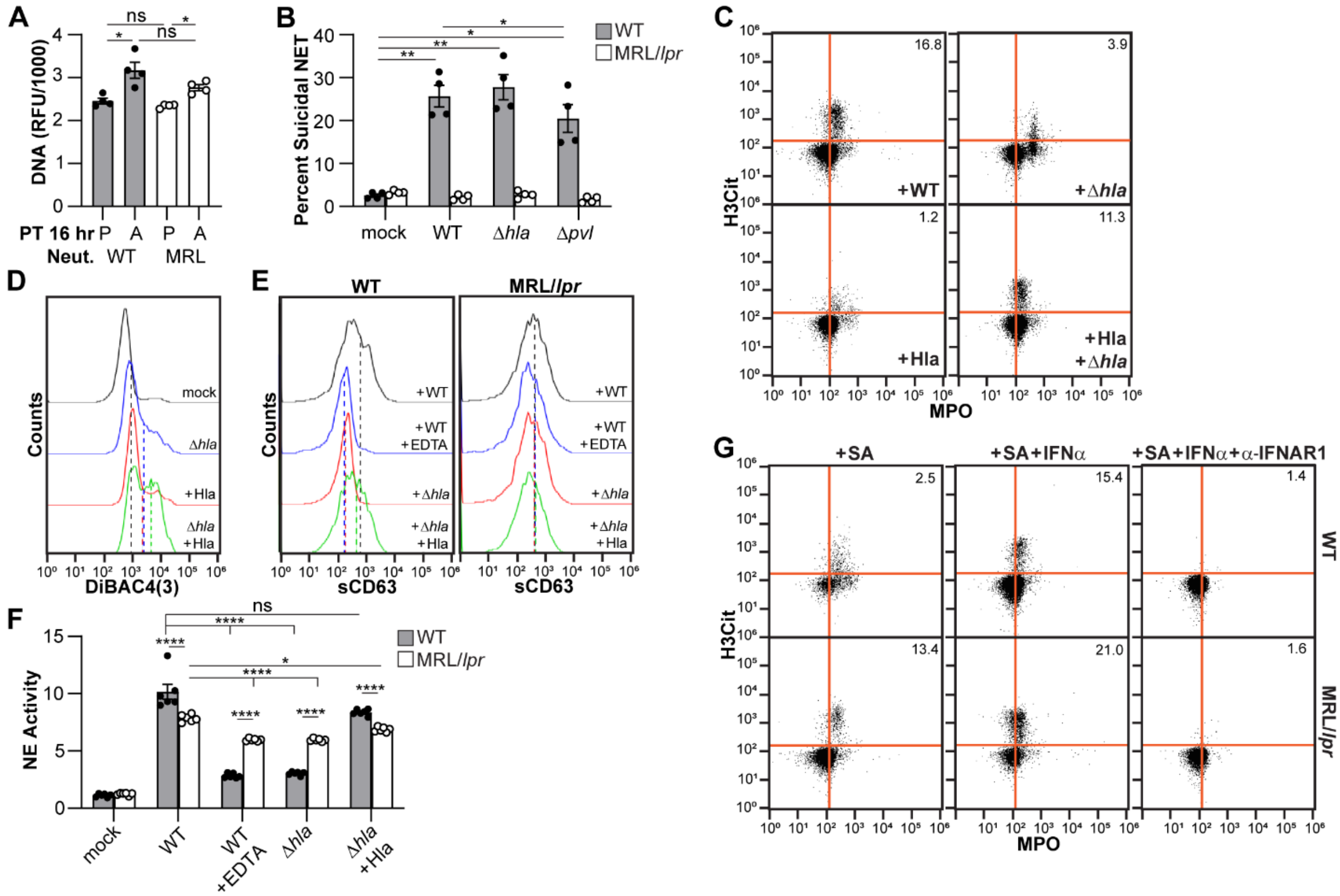

Supplemental Figure 3: Hla expression does not affect suicidal NETosis. (**A**) WT and MRL/*lpr* neutrophils were treated with PMA or A23187 for 16 hours and the resulting NETs subsequently stained to quantify DNA. (**B**) WT or MRL/*lpr* neutrophils were cultured with WT, Δ*hla*, Δ*pvl,* or *Δhla* + *hla S. aureus* in the presence or absence of Hla protein and suicidal NETosis was quantified after 3 hours by flow cytometry. (**C**) WT neutrophils were cultured with WT or Δ*hla S. aureus* in the presence or absence of Hla protein and vital NET release was quantified after 30 minutes by flow cytometry. (**D**) WT neutrophils were loaded with $DiBAC_4(3)$ and stimulated with Δ*hla* and/or Hla protein. The median fluorescence intensity (MFI) was quantified by flow cytometry and normalized to unstimulated neutrophils. (**E-F**) WT or MRL/*lpr* neutrophils were cultured with WT or Δ*hla S. aureus* in the presence or absence of EDTA or Hla protein. After 30 minutes, (*E*) neutrophils were stained for surface expression of CD63 (primary granules) and quantified by flow cytometry or (*F*) supernatants were isolated and NE activity quantified using a fluorescence plate reader. (**G**) WT or MRL/*lpr* neutrophils were treated with IFNα, and/or an α-IFNAR1 antibody and cultured with *S. aureus*. After 3 hours, vital NET release was quantified by flow cytometry. (*A-B*) Each point represents neutrophils isolated from a single mouse. (*A*) One- or (*B*) two-way ANOVA with Tukey multiple comparisons test; error bars reflect standard error (*$p \leq 0.05$, **$p \leq 0.01$, ns = not significant). (*D-E*) Dashed line = MFI.

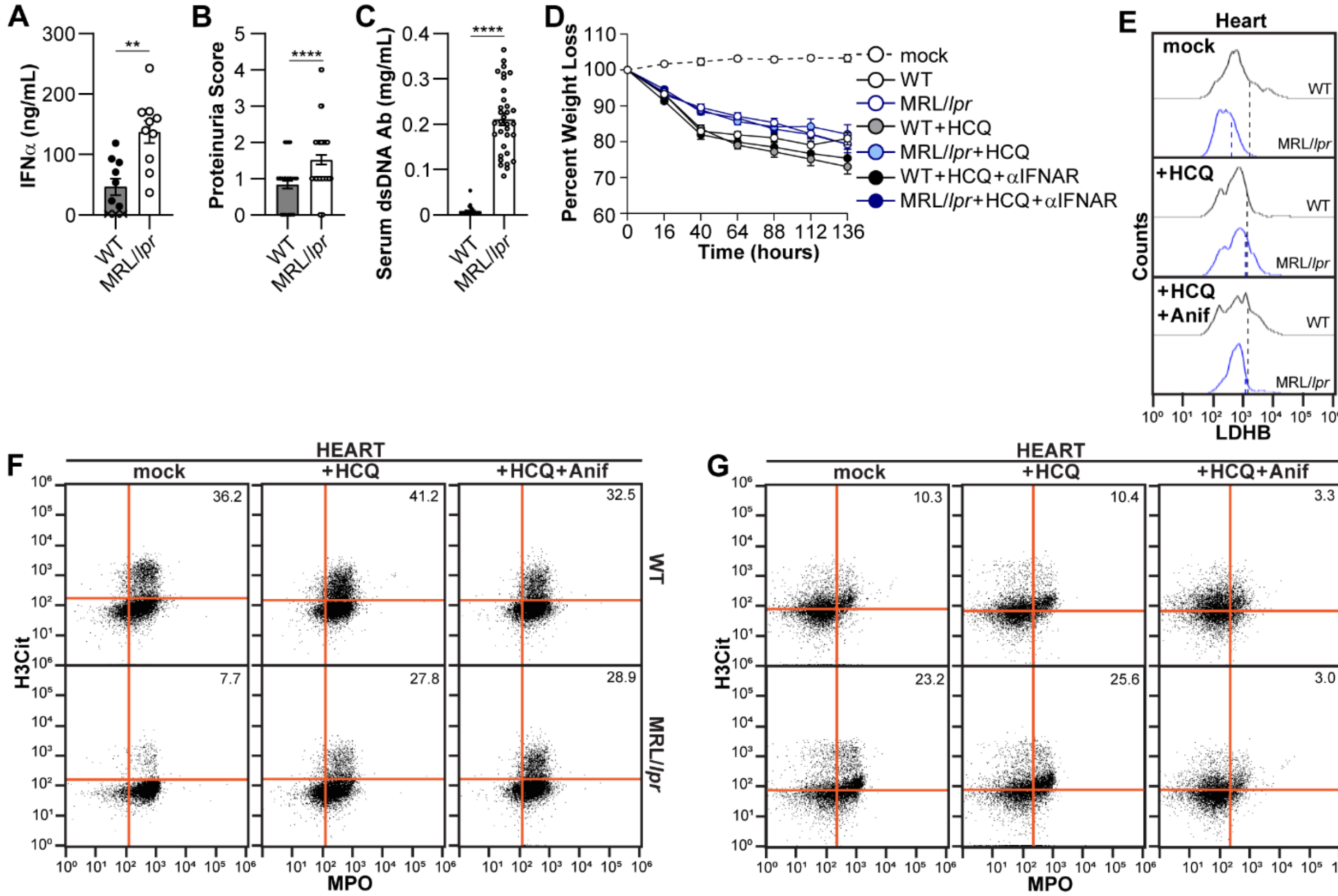

Supplemental Figure 4: MRL/*lpr* have increased IFNα during infection. WT or MRL/*lpr* mice were retro-orbitally infected with *S. aureus* and (**A**) 6 days post-infection, serum was isolated and IFNα quantified by ELISA. (**B**-**C**) The day before infection, (*B*) proteinuria scores were assigned by quantifying protein levels in the urine (0=Trace, 1=30 mg/dL, 2=100 mg/dL, 3=300 mg/dL, 4=2000+ mg/dL) and (*C*) IgG levels were quantified from the serum of the mice by ELISA. (**D**) Weight loss was monitored during infection. Percent weight loss is normalized to starting weight. Neutrophils from the heart were analyzed for (**E**) LDHB protein expression, (**F**) suicidal NETosis, and (**G**) vital NET release by flow cytometry. (*A*-*C*) Each point represents a single mouse or (*D*) the mean of all mice. (*A*-*C*) Unpaired t test; (*A*-*D*) error bars reflect standard error (**p ≤ 0.01,****p ≤ 0.0001). (*E*-*F*) Dashed line = MFI.

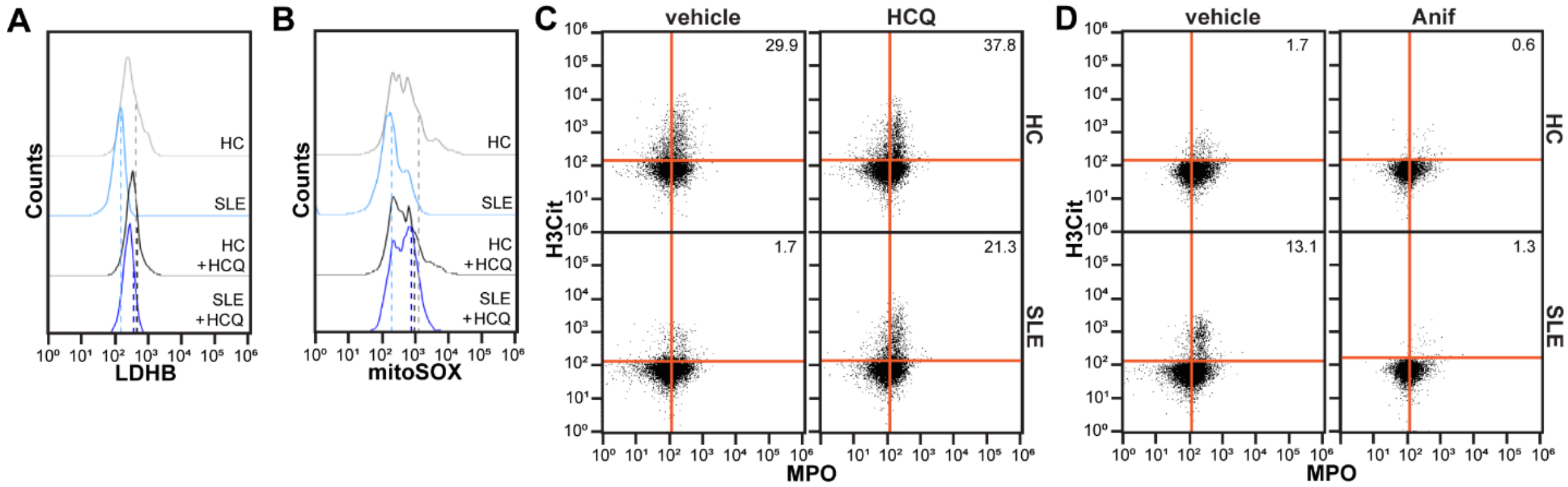

Supplemental Figure 5: HCQ and anifrolumab restore NET subtype regulation in SLE neutrophils. Neutrophils were isolated from HC or SLE patients. (**A**) LDHB protein expression in unstimulated neutrophils or, (**B**) mitochondrial ROS, (**C**) suicidal NETosis, (**D**) vital NET release in response to *S. aureus* were quantified by flow cytometry. (*A*-*B*) Dashed line = MFI.

	ID	Age (yrs.)	Patient sex	Race	Weight (pounds)	Lupus criteria score	Total SLICC Score	Total SLEDAI-2K Score	Creatinine level (mmol)	Protein	dsDNA result	C3 level	C4 level
Prior Study (Wise et. al)	SLE1	20-24	2	1	304	22	0	3	100.2	6.6	1	106	26
	SLE2	40-44	2	1	219.8	42	4	20	110.2	191.9	>300	74	7.9
	SLE3	30-34	2	1	143.2	30	2	13	155.1	74.3	<1	109	13.4
	SLE4	35-39	1	2	253	40	8	0	116.4	36.5	3	109	15.4
	SLE5	65-69	2	2	194.8	10	5	0	48.3	75	3	123	27
	SLE6	40-44	2	1	191	33	2	3	213.1	416.4	10	125	34.2
	SLE7	20-24	1	2	222.6	31	2	8	216.5	22.1	15	136	35.5
	SLE8	35-39	2	1	231	31	6	1			238	117	23.9
	SLE9	40-44	2	1	214.2	43	4	1	292.4	81	295	92	16.2
	SLE10	50-54	1	1	136.8	13	1	0		5.8	5	92	21.9
	SLE11	60-64	2	2	183.6	10	2	0	120.8	9.7	<1	95	19.5
This Study	SLE12	25-29	2	2	121	27	2	15	232	42.8	11	85	21.7
	SLE13	30-34	2	1	123.4	16	2	2	289	31.9	<1	121	16.9
	SLE14	60-64	2	2	190	10	6	0					
	SLE15	40-44	2	1	229	16	2	0	217.9	91.9	78	140	25.1
	SLE16	60-64	2	1	171.8								
	SLE17	45-49	2	1	248.6	31	16	4			3	148	22.8
	SLE18	25-29	2	2	143.2	24	0	4	36.6	3	23	85	6.5
	SLE19	20-24	1	2	240.4	31	1	2					
	SLE20	30-34	2	2	182	18	0	4	118.4	103.6	5	111	6.4
	SLE21	30-34	2	1	137.2	21	1	2			<1	116	13.5
	SLE22	30-34	2	1	118.6	15	0	4			19	103	11.3
			1= Male<br>2 = female	1= Black<br>2 = White-Non-Hispanic									

Supplemental Table 1: SLE donor demographics and disease state.

	ID	Age (yrs.)	Weight (pounds)	Hydroxychloroquine (Plaquenil) units taken daily (e.g. 15mg's) daily	Mycophenolate mofetil (Cellcept) units taken daily	Azathioprine (Imuran) daily dose	Belimumab (Benlysta) daily dose	Tacrolimus daily dose	Voclosporine dosing details	Prednisone dosing details	Anifrolumab dosing details
Prior Study (Wise et. al)	SLE1	20-24	304	200mg twice a day							
	SLE2	40-44	219.8	200mg twice a day	3 g /per day		400mg IV every two weeks			10 mg daily	
	SLE3	30-34	143.2	200 mg twice daily							
	SLE4	35-39	253	200 mg twice a day							
	SLE5	65-69	194.8	200mg twice daily	500 mg three times a day		200mg q week				
	SLE6	40-44	191	200mg twice daily	500mg twice daily						
	SLE7	20-24	222.6	200mg twice daily	1000mg twice daily		10 mg IV monthly				
	SLE8	35-39	231	200 mg twice daily	750 mg twice daily			3 mg twice daily		5mg daily	
	SLE9	40-44	214.2	200mg twice daily	1500 twice daily		400 mg IV every 2 weeks and 200mg sub Q every week				
	SLE10	50-54	136.8	200mg twice daily							
	SLE11	60-64	183.6	200mg twice daily							
This Study	SLE12	25-30	121	200mg once daily			400 mg every two weeks				
	SLE13	30-34	123.4	200mg once daily							300mg every 4 weeks
	SLE14	60-64	190	200mg once daily							
	SLE15	40-44	229	200mg once daily	1500mg daily		200mg Q-week				
	SLE16	60-64	171.8	200mg once daily	1500mg Twice daily						
	SLE17	45-49	248.6	200mg twice daily							
	SLE18	25-29	143.2	200mg twice daily			200mg every week				300mg every 4 weeks
	SLE19	20-24	240.4	200mg twice daily							300mg every 4 weeks
	SLE20	30-34	182	200mg twice daily							300mg every 4 weeks
	SLE21	30-34	137.2	200mg twice daily	3 grams daily						300mg every 4 weeks
	SLE22	30-34	118.6								300mg every 4 weeks

Supplemental Table 2: Medications taken by the SLE donors.
